# The Florida Pancreas Collaborative Next-Generation Biobank: Infrastructure to Reduce Disparities and Improve Survival for a Diverse Cohort of Patients with Pancreatic Cancer

**DOI:** 10.3390/cancers13040809

**Published:** 2021-02-15

**Authors:** Jennifer B. Permuth, Kaleena B. Dezsi, Shraddha Vyas, Karla N. Ali, Toni L. Basinski, Ovie A. Utuama, Jason W. Denbo, Jason Klapman, Aamir Dam, Estrella Carballido, Dae Won Kim, Jose M. Pimiento, Benjamin D. Powers, Amy K. Otto, Jung W. Choi, Dung-Tsa Chen, Jamie K. Teer, Francisca Beato, Alina Ward, Elena M. Cortizas, Suzanne Y. Whisner, Iverson E. Williams, Andrea N. Riner, Kenneth Tardif, Vic Velanovich, Andreas Karachristos, Wade G. Douglas, Adrian Legaspi, Bassan J. Allan, Kenneth Meredith, Manual A. Molina-Vega, Philip Bao, Jamii St. Julien, Kevin L. Huguet, Lee Green, Folakemi T. Odedina, Nagi B. Kumar, Vani N. Simmons, Thomas J. George, Susan T. Vadaparampil, Pamela J. Hodul, J. Pablo Arnoletti, Ziad T. Awad, Debashish Bose, Kun Jiang, Barbara A. Centeno, Clement K. Gwede, Mokenge Malafa, Sarah M. Judge, Andrew R. Judge, Daniel Jeong, Mark Bloomston, Nipun B. Merchant, Jason B. Fleming, Jose G. Trevino, on behalf of the Florida Pancreas Collaborative

**Affiliations:** 1 Department of Cancer Epidemiology, H. Lee Moffitt Cancer Center & Research Institute, Tampa, FL 33612, USA; Kaleena.Dezsi@moffitt.org (K.B.D.); shraddha.vyas@moffitt.org (S.V.); Karla.Ali@moffitt.org (K.N.A.); Toni.Basinski@moffitt.org (T.L.B.); Ovie.Utuama@moffitt.org (O.A.U.); Nagi.Kumar@moffitt.org (N.B.K.); 2 Department of Gastrointestinal Oncology, H. Lee Moffitt Cancer Center & Research Institute, Tampa, FL 33612, USA; Jason.Denbo@moffitt.org (J.W.D.); Jason.Klapman@moffitt.org (J.K.); Aamir.Dam@moffitt.org (A.D.); Estrella.Carballido@moffitt.org (E.C.); DaeWon.Kim@moffitt.org (D.W.K.); Jose.Pimiento@moffitt.org (J.M.P.); benjamin.powers@moffitt.org (B.D.P.); Francisca.Beato@moffitt.org (F.B.); Pamela.Hodul@moffitt.org (P.J.H.); Mokenge.Malafa@moffitt.org (M.M.); Jason.Fleming@moffitt.org (J.B.F.); 3 Department of Public Health Sciences, University of Miami Miller School of Medicine, Miami, FL 33612, USA; aotto@med.miami.edu; 4 Department of Diagnostic Imaging and Interventional Radiology, H. Lee Moffitt Cancer Center & Research Institute, Tampa, FL 33612, USA; Jung.Choi@moffitt.org (J.W.C.); Daniel.Jeong@moffitt.org (D.J.); 5 Department of Biostatistics and Bioinformatics, H. Lee Moffitt Cancer Center & Research Institute, Tampa, FL 33612, USA; Dung-Tsa.Chen@moffitt.org (D.-T.C.); Jamie.Teer@moffitt.org (J.K.T.); 6 Lee Health Regional Cancer Center, Fort Myers, FL 33905, USA; alina.ward@LeeHealth.org (A.W.); bassan.allan@usa.genesiscare.com (B.J.A.); mark.bloomston@usa.genesiscare.com (M.B.); 7 Sylvester Comprehensive Cancer Center, Miller School of Medicine, University of Miami, Miami, FL 33136, USA; ecortizas@med.miami.edu; 8 Cancer Institute, Advent Health Orlando, Orlando, FL 32804, USA; Suzanne.whisner@adventhealth.com; 9 College of Medicine, University of Florida, Gainesville, FL 32610, USA; Iverson.Williams@surgery.ufl.edu (I.E.W.); Andrea.Riner@surgery.ufl.edu (A.N.R.); Jose.Trevino@vcuhealth.org (J.G.T.); 10 Department of Surgery, St. Anthony’s Hospital, St. Petersburg, FL 33705, USA; Ken.Tardif@baycare.org (K.T.); dr.stjulien@baysurgicalspecialists.com (J.S.J.); dr.huguet@baysurgicalspecialists.com (K.L.H.); 11 Tampa General Hospital, University of South Florida, Tampa, FL 33606, USA; vvelanov@health.usf.edu (V.V.); andreask@usf.edu (A.K.); 12 Division of Surgery, Tallahassee Memorial Healthcare, Department of Clinical Sciences, College of Medicine, Florida State University, Tallahassee, FL 32308, USA; wade.douglas@tmh.org; 13 Center for Advanced Surgical Oncology at Palmetto General Hospital, Tenet Healthcare Palmetto General, Hialeah, FL 33016, USA; Adrian.legaspi@TenetHealth.com; 14 Department of Gastrointestinal Oncology, Brian Jellison Cancer Institute, Sarasota Memorial Hospital, Sarasota, FL 34239, USA; kenneth-meredith@smh.com; 15 Lakeland Regional Health, Lakeland, FL 33805, USA; Manuel.Molina@myLRH.org; 16 Department of Surgical Oncology, Mount Sinai Medical Center, Miami Beach, FL 33140, USA; philip.bao@msmc.com; 17 Department of Health Outcomes and Behavior, H. Lee Moffitt Cancer Center & Research Institute, Tampa, FL 33612, USA; Lee.Green@moffitt.org (L.G.); Vani.Simmons@moffitt.org (V.N.S.); Susan.Vadaparampil@moffitt.org (S.T.V.); Clement.Gwede@moffitt.org (C.K.G.); 18 Department of Pharmacotherapy and Translational Research, University of Florida, Gainesville, FL 32610, USA; fodedina@cop.ufl.edu; 19 Division of Oncology, Department of Medicine, University of Florida, Gainesville, FL 32610, USA; thom.george@medicine.ufl.edu; 20 Office of Community Outreach, Engagement, and Equity, H. Lee Moffitt Cancer Center & Research Institute, Tampa, FL 33612, USA; 21 Center for Surgical Oncology, Advent Health Orlando, Orlando, FL 32804, USA; pablo.arnoletti.md@adventhealth.com; 22 Surgery, University of Florida-Jacksonville, Jacksonville, FL 32209, USA; ziad.awad@jax.ufl.edu; 23 Surgical Oncology, University of Florida-Orlando, Orlando, FL 32806, USA; Debashish.bose1@gmail.com; 24 Department of Pathology, H. Lee Moffitt Cancer Center & Research Institute, Tampa, FL 33612, USA; Kun.Jiang@moffitt.org (K.J.); Barbara.Centeno@moffitt.org (B.A.C.); 25 Department of Physical Therapy, University of Florida, Gainesville, FL 32610, USA; smsenf@phhp.ufl.edu (S.M.J.); arjudge@phhp.ufl.edu (A.R.J.); 26 Department of Surgical Oncology, Miller School of Medicine, University of Miami, Miami, FL 33136, USA; nmerchant@med.miami.edu; 27 Division of Surgical Oncology, Department of Surgery, School of Medicine, Virginia Commonwealth University, Richmond, VA 23219, USA

**Keywords:** biorepository, underserved populations, cancer disparities, prospective cohort, pancreatic cancer

## Abstract

**Simple Summary:**

Pancreatic cancer is the third leading cause of cancer deaths and is projected to become the second leading cause by 2030. Striking racial disparities in pancreatic cancer incidence and mortality rates exist nationally and in Florida, with higher rates among African Americans compared to other racial groups. Biological reasons for these disparities remain unexplained, primarily because most pancreatic cancer research has relied on biospecimens and data from Non-Hispanic Whites. Multidisciplinary teams from fifteen hospitals throughout the state of Florida have partnered together and with patients newly-diagnosed with pancreatic cancer to build the first state-wide biobanking infrastructure we know of that is dedicated to reducing the disproportionate burden of pancreatic cancer affecting African Americans. We describe important information on ascertainment and recruitment strategies and standard operating procedures developed to collect, process, store, and transfer biospecimens, medical images, and data from a diverse cohort of participants. The infrastructure described in this manuscript is intended to serve as a strong foundation for further research into biological, behavioral, socioeconomic, and environmental factors that may contribute to observed disparities and a starting point to develop interventions to tackle these factors. This multi-institutional infrastructure can serve as a prototype for the development of similar resources across the country and disease sites.

**Abstract:**

*Background*: Well-annotated, high-quality biorepositories provide a valuable platform to support translational research. However, most biorepositories have poor representation of minority groups, limiting the ability to address health disparities. *Methods*: We describe the establishment of the Florida Pancreas Collaborative (FPC), the first state-wide prospective cohort study and biorepository designed to address the higher burden of pancreatic cancer (PaCa) in African Americans (AA) compared to Non-Hispanic Whites (NHW) and Hispanic/Latinx (H/L). We provide an overview of stakeholders; study eligibility and design; recruitment strategies; standard operating procedures to collect, process, store, and transfer biospecimens, medical images, and data; our cloud-based data management platform; and progress regarding recruitment and biobanking. *Results*: The FPC consists of multidisciplinary teams from fifteen Florida medical institutions. From March 2019 through August 2020, 350 patients were assessed for eligibility, 323 met inclusion/exclusion criteria, and 305 (94%) enrolled, including 228 NHW, 30 AA, and 47 H/L, with 94%, 100%, and 94% participation rates, respectively. A high percentage of participants have donated blood (87%), pancreatic tumor tissue (41%), computed tomography scans (76%), and questionnaires (62%). *Conclusions*: This biorepository addresses a critical gap in PaCa research and has potential to advance translational studies intended to minimize disparities and reduce PaCa-related morbidity and mortality.

## 1. Introduction

Pancreatic cancer (PaCa) is the deadliest malignancy in the United States (US), with a five-year relative survival rate of 10% [1]. Surgical resection offers the only chance for long-term survival, but only 15–20% of cases are resectable at diagnosis. Due to the lack of effective strategies for prevention, early detection, and treatment, PaCa is projected to become the second leading cause of cancer deaths by 2030 [2]. Coinciding with the rise in the number of PaCa diagnoses and deaths is a notable health disparity [2,3,4,5,6,7,8,9,10,11,12], with African Americans (AA)/Blacks having significantly higher PaCa incidence and mortality rates compared to Non-Hispanic White (NHW) and Hispanic/Latinx populations (H/L) [3].

Florida has the third largest population in the US, is home to more than 3.5 million AA [13] and 6.1 million H/L [14] and is surpassed only by California in lives lost to PaCa annually [1]. In 2020, 3570 (7.9%) of the 45,300 cancer-related deaths among Floridians will be due to PaCa and occur mainly in NHW, AA, and H/L [1], yet PaCa disparities research is limited in Florida’s diverse population. We used Florida Cancer Data System (FCDS) Registry data [15] to estimate age-adjusted PaCa incidence and mortality rates and found that AA had the highest incidence and mortality rates across genders, mirroring national disparities [16]. 

Unfortunately, most PaCa disparities research has been descriptive, with inequities unexplained by epidemiologic and socioeconomic factors or access barriers [2,3,4,5,6,7,8,9,10,11,12,17]. Compared to NHW, AA are less likely to: be referred to PaCa specialists, be diagnosed/treated at high-volume hospitals, receive surgery or chemotherapy, or be insured [11,18,19,20]. Structural racism at individual and institutional levels may also drive racial health inequities [21]. Biological factors may also contribute to PaCa disparities, but the lack of well-annotated biospecimens from AA have precluded advances in this area. 

Biorepositories provide a rich platform to study and address cancer health disparities and improve outcomes. High-quality, well-annotated national [22] and institutional [23] biorepositories have been developed to study PaCa and related conditions (chronic pancreatitis and diabetes [22]), but addressing health disparities was not a focus in their development and biospecimens from minority groups are scarce in these resources. Our objective is to study and address PaCa disparities by building a robust state-wide ‘next-generation biobank’ containing viable tissues, biofluids, images, and data with a racially/ ethnically diverse cohort of Floridians with PaCa and its precursors. 

## 2. Materials and Methods

### 2.1. Participating Sites, Multidisciplinary Expertise, and Advisory Boards

**Figure 1:**
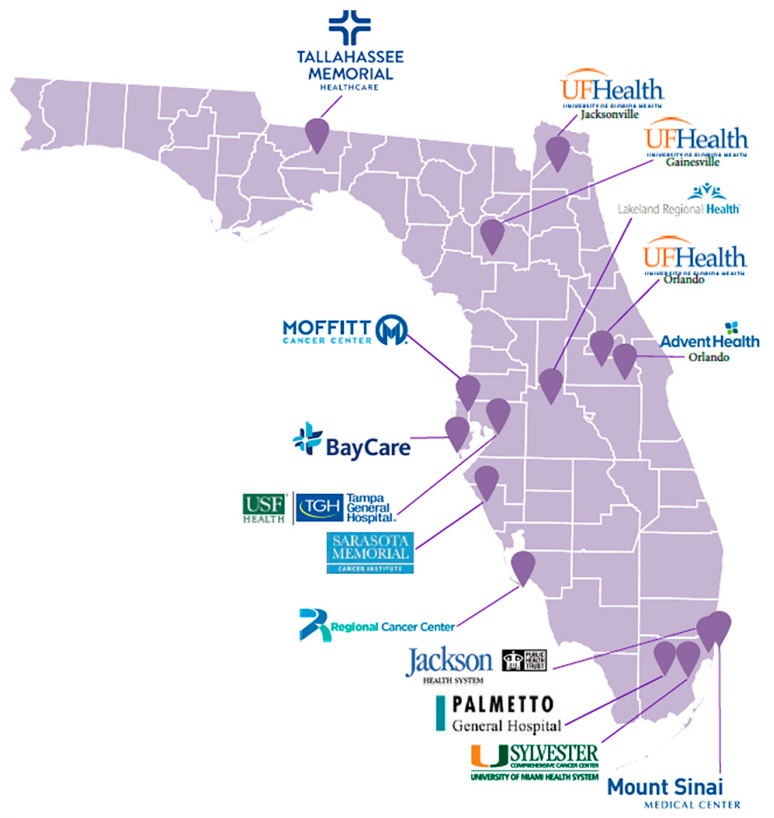
Florida Pancreas Collaborative Study Sites.

The Florida Pancreas Collaborative (FPC) was founded in 2015 by investigators (JBP, MPM, JGT, NBM) from the three main academic cancer centers based in Florida: Moffitt Cancer Center and Research Institute (MCC, Tampa), the University of Florida Health Cancer Center (UFG, Gainesville), and the Sylvester Comprehensive Cancer Center/University of Miami (UOM, Miami) [24]. Florida Agency for Health Care Administration (AHCA) [25] inpatient discharge data was used to identify institutions throughout Florida with the highest numbers of AA, H/L, and NHW individuals diagnosed and treated for PaCa. We then used internet queries and our professional network to identify and contact clinicians (primarily surgeons and oncologists) to assess interest in participation. Requirements for participation included having a dedicated site principal investigator (PI), institutional support/backing, and willingness to contribute to a common biorepository using standard operating procedures (SOPs). With grant funding from the State of Florida’s James and Esther King Biomedical Research Program in 2018, the FPC expanded to include twelve additional institutions (academic and community cancer centers and private hospitals) including (in alphabetical order): Advent Health Orlando (AHO, Orlando, FL, USA), Jackson Memorial Hospital (JMH, Miami, FL, USA), Lakeland Regional Health Hollis Cancer Center (LRH, Lakeland, FL, USA), Mount Sinai Medical Center (MSM, Miami, FL, USA), Palmetto General Hospital (PGH, Hialeah, FL, USA), Regional Cancer Center (RCC, Fort Myers), Saint Anthony’s Hospital/BayCare (STA, St. Petersburg, FL, USA), Sarasota Memorial Hospital (SMH, Sarasota, FL, USA), Tallahassee Memorial Healthcare (TMH, Tallahassee, FL, USA), the University of Florida Health (UFJ, Jacksonville, FL, USA), Orlando Health University of Florida Health Cancer Center (UFO, Orlando, FL, USA), and the University of South Florida/Tampa General Hospital (USF, Tampa, FL, USA). MCC serves as the lead coordination/management center. A map of participating FPC sites is displayed in Figure 1.

Our team has expertise in disciplines including molecular epidemiology, surgical oncology, radiology, gastrointestinal endoscopy, medical oncology, radiation oncology, nutrition, genetics, molecular biology, muscle physiology, behavioral science, pathology, biostatistics, and bioinformatics. A scientific advisory board advises, oversees, and evaluates activities related to study aims and consists of members chosen for their research and clinical expertise and strong regional and national leadership in the areas of Diversity/Health Equity Research, PaCa Treatment, Nutrition/Cachexia Research, and Tobacco Cessation. Given that recruiting individuals to participate in biobanks can be challenging, we are working with community partners including MCC’s National Cancer Institute (NCI)-funded Tampa Bay Community Cancer Network and the Geographic Management of Cancer Health Disparities Program, the George Edgecomb Society, and local affiliates of the Pancreatic Cancer Action Network to expand our community advisory board which includes PaCa survivors and advocates. 

### 2.2. Study Population

To be eligible for participation, an individual of any gender identity must: be at least 18 years of age; self-report as NHW, AA, or H/L; present to the gastrointestinal (GI) clinic, surgery, or endoscopy at a participating site with a strong clinical suspicion or diagnosis of a pancreatic tumor based on symptoms, imaging, biopsy, and/or blood-work; have a treatment-naïve pancreatic tumor at the time of enrollment; be able to understand and voluntarily sign the informed consent; and be willing to complete study questionnaire(s) and donate medical images and biospecimens during standard-of-care (SOC) procedures. Confirmation of the diagnosis is sought for all cases and is typically confirmed by pathologic review of tissue obtained through routine diagnostic procedures by a site pathologist. We also rely on cancer registry data and the electronic medical record (EMR). To increase the breadth of cases for inclusion, we enroll patients with operable or inoperable exocrine and endocrine pancreatic cancers (including pancreatic ductal adenocarcinomas (PDAC) and pancreatic neuroendocrine tumors (PNET)), and patients with pre-malignant cysts including intraductal papillary mucinous neoplasms (IPMN) and mucinous cystic neoplasms (MCN).

### 2.3. Study Design

This research infrastructure grant uses a prospective longitudinal multi-institutional cohort design. The project began administratively in May of 2018, and a 10-month run-in period was essential for infrastructure-building. With input from stakeholders, substantive accomplishments during the run-in period included: development of a study logo, recruitment materials, and study web-site; finalizing a uniform study protocol, informed consent document, questionnaires, and data collection instruments/case report forms (CRF); translation of study materials into Spanish; obtaining regulatory approval through the single Institutional Review Board (sIRB; Advarra, Inc., Columbia, MD, USA), development of SOPs for data, image, and biospecimen collection, processing, storage, and transfer; building a centralized platform for data collection, management, and workflow; and hiring and training staff. Resources collected at each time-point (baseline, 6 months, and 12 months) are summarized in Table 1. 

### 2.4. Ascertainment and Recruitment Strategies

Engagement of the entire clinical research team is critical to successfully building a biobank. Each participating site has a lead coordinator who works closely with the site PI and the program manager at MCC. This coordinator is integrated into each clinic’s workflow and is responsible for screening daily clinic and procedure logs to identify individuals to approach regarding participation. To aid in recruitment, we developed a study-specific flyer, informational brochure, and public-facing web-site (www.floridapancreascollaborative.org) (4 January 2021) with a members-only portal accessible via secure sign-in. Additionally, based on data published by our team and others supporting incentives as motivating factors in increasing study participation [26,27,28,29], participants receive compensation (in the form of gift card(s)) to Amazon or Walmart) as a token of appreciation for their time and effort upon completion of baseline ($10) and follow-up questionnaires ($5 each). 

### 2.5. Overview of Study Workflow and Data Management/Tracking

A customized cloud-based data management/engagement platform was built in partnership with DatStat, Inc. (Seattle, WA, USA). This platform helps sites efficiently assign unique identification numbers, assess eligibility, obtain informed consent electronically, administer questionnaires, and track biospecimens (Figure S1). Detailed views of the platform and select components are displayed in Figures S2 and S3. The platform also manages and stores study-related data and enables queries. Study sites may only access information pertaining to their own participants while MCC has regulatory approval to access and analyze data across sites.

### 2.6. Data Collection Procedures and Instruments

At each timepoint, the coordinator records the participant’s height and weight, measures their waist and hip circumference, and administers a 3-page health screen (Table 1). The health screen evaluates the presence of conditions prevalent among patients with PaCa: cancer cachexia, a progressive and debilitating muscle-wasting syndrome characterized by unintentional weight loss, muscle atrophy, fatigue, and limited tolerance of chemotherapy; depression and distress; and past and present smoking history. The health screen comprises the abridged version of the Patient-Generated Subjective Global Assessment, a revised version of the Edmonton Symptom Assessment System, and the Canadian Problem Checklist (Figure S4). This screen helps providers better understand participant concerns and ‘flags’ issues via a customized ‘Health Screen’ report to proactively enhance patient care, outcomes, and experiences through education/counseling and referral to other professionals (i.e., dieticians, physical therapists/rehabilitation, psychiatrists, social workers) as needed. An example of a Health Screen Report is shown in Figure S5. 

Participants complete online- or teleform-based questionnaires that solicit core demographic, clinical, epidemiologic and exposures such as tobacco use (Table 2). The questionnaire also contains several validated instruments to assess mental health, sleep, nutrition, physical activity, lifetime exposure to acute and chronic stressors, self-reported symptoms, and quality of life. Validated instruments used in the health screen or baseline questionnaire are listed in Table 3. This self-reported data is supplemented by data abstracted from the EMR into case report forms (CRF) and data requested from the FCDS. The CRFs capture presenting signs, symptoms, and comorbidities; pre-treatment and treatment details; and follow-up information (Table 2). For data management and tracking, blood, tissue, and image collection/transfer CRFs are also used.

### 2.7. Biospecimen Collection, Processing, and Storage

Since differences in biospecimen collection, processing, and storage methods can confound findings in biomarker studies, SOPs were developed and tested at MCC and modified to ensure compliance at participating sites. Additionally, all supplies and reagents are provided by MCC. Digital bar-code labeled (DBL) cryogenic vials were chosen for long-term storage/preservation because of their durability and ability to facilitate accurate data entry and rapid retrieval with a unique sample ID not linked to patient identifiers. Freezers at MCC have autonomous continuous temperature monitoring and an alarm system to notify responsible parties of malfunction. Freezers are on a stable power grid with backup generators and are above ground level to prevent flood damage.

#### 2.7.1. Blood

**Figure 2:**
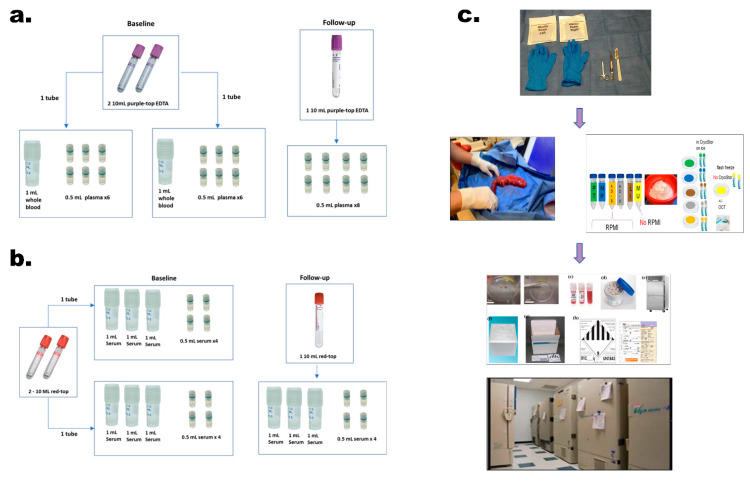
Blood and Tissue Collection and Processing Workflow. (**a**) Purple-topped EDTA tubes are processed and stored at Baseline and Follow-up time-points into whole blood (Baseline) and plasma (Baseline and Follow-up). (**b**) Red-topped tubes are processed and stored at Baseline and Follow-up timepoints into serum. (**c**) During the surgical procedure, a debridement kit containing sterile supplies (gloves, gauze, scissors, forceps, scalpel, and drape) is open in preparation for receiving tissue samples. Collected tissue samples are placed in labelled conical tubes on ice with 20mL RPMI 1640/2% penicillin-streptomycin (Pancreatic Tumor (PT), Normal Pancreas (NP), Adipose–omental (AD-O), Adipose–subcutaneous (AD-S), and Liver (LI), or in an empty conical tube Muscle (MU). Samples are then transferred to labelled petri dishes for mincing. Non-muscle tissues are placed in cryovials with CryoStor CS10 to slow freeze in a Mr. Frosty overnight prior to long-term storage in liquid nitrogen. Muscle tissues are either placed in cryovials with no media and snap frozen prior to long-term storage in a −80 °C freezer (Step A) or frozen in isopentane, embedded in OCT, and stored in a cassette in a −80 °C freezer until shipment and future analysis (Step B).

Peripheral blood is donated at baseline (± 30 days of the diagnosis date) and at follow-up in conjunction with routinely-scheduled venipuncture. Using SOPs in line with NCI’s Best Practices, four 10 mL tubes (two red-top, two purple-top EDTA) are collected at baseline and two 10 mL tubes (one red-top, 1 EDTA) are requested at follow-up. The date and time samples are drawn, processed, and stored, and details such as visual hemolysis assessment are recorded. EDTA tubes are slowly inverted 8–10 times and then transferred to the institution’s laboratory for processing 30 min to 2 h after collection. For samples collected at baseline, 1 mL of whole blood (from each EDTA tube) is aliquoted into a cryovial and remaining blood is processed for plasma by centrifugation at 1300 *g*/RT/10 min and aliquoted as in Figure 2a. The red-topped tubes are processed for serum after allowing 30 min for clotting by centrifuging @1300 *g*/RT/10 min. At baseline, serum is aliquoted in 1 mL and 0.5 mL volumes (Figure 2b). Follow-up cryovial specifics are shown in Figure 2a,b. All samples are stored in cryoboxes at −80 °C until transferred to MCC. 

#### 2.7.2. Surgically-Resected Tissue

At resection, sites collect and process pancreatic tumor (PT), normal pancreas (NP), muscle (MU) from the upper right quadrant of the rectus abdominus, adipose-subcutaneous (AD-S) above rectus muscle and adipose-omental/intraperitoneal (AD-O) tissue, and sites of metastasis such as liver (LI). A debridement kit containing sterile supplies (gloves, drape, forceps, scissors, scalpel, and gauze) is provided for use in the pathology gross room (Figure 2c). Sites organize supplies with color-coded cryodots corresponding to each tissue type including pre-labeled 50 mL conical tubes containing 20 mL RPMI 1640/2% penicillin-streptomycin (p/s), petri dishes, and 2 mL cryovials (Argos Polarsafe Cryogenic Storage Vials with External Cap, Cole-Parmer, Vernon Hills, IL, USA), filled with 1 mL of CryoStor CS10 freezing solution (BioLife Solutions, Inc., Bothell, WA, USA) for all tissue types except for MU. (MU is not placed into RPMI or CryoStor). For each tissue type, the time of tissue removal, time received in pathology suite (for PT & NP), time placed in media, and time of freezing is recorded. After tumor resection, the time until immersion into preservative is under 30 min to minimize ischemia and degradation.

When the resected pancreatic tumor specimen is removed, the surgeon obtains an en face section of the pancreatic transection margin for frozen-section and the specimen is transported on wet ice for gross analysis by a pathologist and/or pathology assistant. The local pathology team determines whether ample pancreatic tumor tissue is available for diagnosis and cancer staging and whether a portion of the specimen can be banked without disrupting the accuracy of the pathology reporting for patient care. The priority for banking is the central area of the tumor followed by the tumor margin, and a minimum of a 5.0 mm^3^ tumor fragment from the epicenter is requested. Upon obtaining a negative margin on the pancreatic edge, we obtain a “normal” pancreatic biopsy at the reconstructive end. The site priority is (in decreasing order): distant pancreas, grossly uninvolved pancreas, or perilesional uninvolved pancreas (normal tissue adjacent to the tumor or surrounding stroma). Non-MU tissue samples are placed in the corresponding conical tube on ice and transferred to a designated institutional laboratory/processing facility where sterile forceps are used to transfer each tissue type to the corresponding pre-labeled petri dish with 3 mL of RPMI with 2% p/s from each sample’s conical tube. Non-muscle samples (PT, NP, AD-S, AD-O and any metastatic specimens) are minced into 2–3 mm^3^ fragments using sterile forceps and scalpels and 2–6 fragments are transferred to pre-labeled cryovials preloaded with 1 mL CryoStor. Cryovials are immediately stored at 4 °C for 30 min to allow CryoStor to penetrate the tissue and then placed into a Mr. Frosty Freezing Container (ThermoFisher Scientific, Pittsburgh, PA, USA) (4 °C) and stored at −80 °C overnight. Cryovials are transferred to a liquid nitrogen (LN2) storage unit, vapor phase or shipped to MCC the next day (Figure 2c), and stored for future research.

Two main steps are involved in processing MU tissue: flash freezing for biochemical analysis (step A) and embedding in optimal cutting temperature (OCT) compound for morphological assessment (step B). Upon incision through the skin and dissection through subcutaneous fat, a 2.0 × 1.0 cm muscle biopsy specimen is obtained and sharply divided into four fragments, avoiding cautery burns. Three of the 4 fragments are placed into pre-labeled cryovials and put in a LN2-containing dewar (or dry ice/ethanol slurry if LN2 is not available) and stored at −80 °C for step A. The fourth fragment (for step B) is wrapped in gauze pre-moistened with ice-cold PBS, placed in a pre-labeled conical tube on wet ice, embedded in OCT, frozen in LN2-cooled isopentane, cooled in LN2, and stored at -80°C until shipped on dry ice to the UFG site for storage and future analysis by our muscle physiologists (A.R.J., S.M.J.). 

#### 2.7.3. Endoscopic Fine Needle Aspirate and Core Biopsies

In September of 2019, we began collecting cystic fluid and tissue from patients undergoing endoscopic ultrasound-guided fine needle aspirate (FNA) and fine needle aspirate biopsies (FNAB) from cystic and solid pancreatic neoplasms, respectively. Initial passes are designated for diagnostic purposes, and up to three additional passes are collected for research if deemed safe by the endoscopists. Residual cystic fluid over 2 mL is aliquoted into 4 × 1 mL digital barcode-labeled cryovials with 0.5 mL of sample/cryovial and stored at –80 °C. FNAB smears and cell blocks are prepared according to institutional cytology laboratory standards. The FNAB needle is rinsed in 5–10 mL of balanced salt solution or other medium, the sample is centrifuged, and the pellet is used to prepare a cell block. The residual supernatant is saved and stored at −80 °C until shipped to MCC.

### 2.8. Repository of CT Images

Consistent with the missions of NCI’s Quantitative Imaging Network and Cancer Imaging Archive, we use best practices for acquisition, de-identification, curation, and secure transmission/sharing of pancreas-specific radiologic images with focus on CT scans. SOPs for Digital Imaging and Communications in Medicine (DICOM) images and metadata that are being followed [30]. Participating sites provide CT scans to MCC’s Quantitative Imaging (QI) Team via CD or electronically to a secure ShareFile or Powershare portal. Instructions for DICOM header re-labeling and transmission are followed. Upon receipt, the QI Team uploads the corresponding imaging report and CT scans into MCC’s Healthmyne Research Infrastructure and logs scan details into an Excel database. Additional metrics are abstracted and entered into a standardized template for radiologic reporting of PDAC [31]. Images are also being used to perform body composition analyses.

### 2.9. Development of an Integrated and Centralized Virtual Data Repository

A central database linking individual-level de-identified data to biospecimens and images across internal and external source systems has been created and maintained by MCC and is known as the Florida Pancreas Collaborative Data Repository (FPCDR). Outside of the demilitarized zone (DMZ), the virtual repository integrates electronic survey data ascertained through DatStat with paper survey data provided through MCC’s Participant Research, Interventions, and Measurement (PRISM) Core, data from the online Stress and Adversity Inventory (STRAIN) tool [32] housed on a server at the University of California, and cancer registry data from the FCDS (Figure S6). The study website and the ShareFile application is also housed outside the DMZ until joined to participant information and stored on the MCC network as part of the image repository, DatStat database, or other source systems. For example, demographic data is transferred using HL7 to MCC’s Clinical Trails Management System (Oncore), and biospecimen-level annotation is transferred into LabVantage and includes variables such as the date of collection, tissue of origin, histological diagnosis, storage format (i.e., LN2, OCT, −80 °C), the number of cryovials of each sample and their location. Reporting and querying functions are used to generate summary reports and ad hoc queries. In addition to the security in place through the DatStat platform (which include including Health Insurance Portability and Accountability Act (HIPAA) compliance, 21 CFR Part 11 Compliance, SOC 2 Type 2 Certification, and Privacy Shield certification), numerous safeguards have been incorporated into the centralized repository to maintain patient confidentiality and ensure HIPAA compliance. Data also undergo quality and post-load checks to identify incorrect or missing data.

### 2.10. Descriptive Statistics 

Frequencies and percentages were generated for categorical variables and means and standard deviations (SD) were calculated for continuous variables. Distributions of covariates were compared across racial/ethnic groups using chi-squared tests, fisher’s exact tests, t-tests, and generalized linear models. *p* Values < 0.05 were considered statistically significant. Analyses were performed using SAS version 9.4 (SAS Institute, Cary, NC, USA). 

## 3. Results

### 3.1. Enrollment

**Figure 3:**
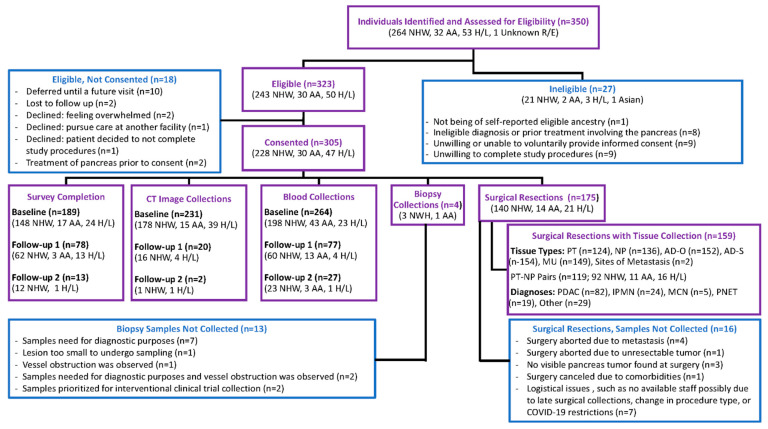
Summary of Recruitment, Survey Data, Image, and Biospecimen Collection Efforts to Date, by Race/Ethnicity. The flow diagram depicts the number of individuals eligible and ineligible for the study, as well as number of consented participants who have donated biospecimens, computed tomography (CT) images, and completed surveys. Abbreviations: Non-Hispanic White (NHW), African American (AA), Hispanic/Latinx (H/L), Pancreatic Tumor (PT), Normal Pancreas (NP), Adipose–omental (AD-O), Adipose–subcutaneous (AD-S), Muscle (MU), Pancreatic Ductal Adenocarcinoma (PDAC), Intraductal Papillary Mucinous Neoplasm (IPMN), Mucinous Cystic Neoplasm (MCN), Pancreatic Neuroendocrine Tumor (PNET).

Recruitment was initiated at MCC in March 2019. As of August 2020, 13 of 15 FPC sites were actively recruiting with 2 sites delayed due to finalizing the site agreement (*n* = 1) and staff turnover (*n* = 1). A total of 350 individuals (264 NHW, 32 AA, 53 H/L and 1 unknown race/ethnicity) were identified and assessed for eligibility to participate. Of 323 individuals deemed to be eligible (243 NHW, 30 AA, and 50 H/L), 305 enrolled (228 NHW, 30 AA, and 47 H/L), with participation rates of 94%, 100%, and 94%, respectively. Nearly 41% (*n* = 124) of enrolled participants were recruited at sites other than Florida’s three academic cancer centers (MCC, UFG, UOM) (Figure S7). Moreover, participants have been recruited from all fifteen counties in the coordinating center’s catchment area and from 69% of all Florida counties. A detailed flowchart regarding recruitment outcomes is shown in Figure 3.

### 3.2. Study Population

Select study population characteristics are given in Table 4. The average age at diagnosis is 68 years, with AA and H/L diagnosed significantly younger than NHW (64 and 63 versus 70 years, respectively, *p* = 0.0001). Most participants (*n* = 161, 53%) are female, with the highest proportion observed among H/L. Education, income level, and health insurance status did not significantly differ between racial/ethnic groups. The most common presenting symptoms included weight loss > 5% over the past 6 months (*n* = 115, 40.9%), abdominal pain (*n* = 100, 49.8%), and fatigue (*n* = 98, 34.7%). Mean BMI was highest among AA (28 kg/m^2^) followed by NHW (27 kg/m^2^) and H/L (25 kg/m^2^). Almost one-third of the study cohort (*n* = 90) reported a personal history of diabetes, with no significant differences between racial/ethnic groups. Most participants have a confirmed diagnosis of PDAC (*n* = 183, 61.4%), followed by IPMNs and PNET, each representing 11.7% (*n* = 35) of cases. Seven of the AA cases with confirmed histology (25%) had PNETs, which is higher than the proportion of PNETs in NHW (11.1%) and H/L (6.5%). Compared to PDAC cases, PNET cases were: more likely to be diagnosed younger (63 vs. 69 years) and less likely to have presented with jaundice or >5% weight loss (data not shown). Of the 183 PDAC cases, staging data is available for 99 (60 stage I/II; 39 stage III/IV). Of 22 PNET cases with staging data, 54.3% are stage I/II. Overall, based primarily on self-reported outcomes in the health screen and baseline questionnaire, the prevalence of cachexia, depression, former tobacco use, and current tobacco use at study enrollment were 32.9%, 35.1%, 43.6%, and 12.1% respectively.

### 3.3. Exited Participants

Forty-seven participants have been exited or withdrawn from the study thus far. The notation of “exited” ensures that reminder emails to participants and study sites regarding follow-up tasks for the participant are terminated, but the data compiled during active participation is retained for future analyses. Reasons for exiting include death (*n* = 28), screen failure/ineligible (*n* = 8), withdrawal by the participant (*n* = 6) or physician (*n* = 1), treated at another institution (*n* = 3), or admission to hospice (*n* = 1). Of those who died, most (*n* = 25, 89.2%) had PDAC, 2 had chronic cholecystitis, and 1 has an unknown diagnosis. Sociodemographic characteristics of exited and non-exited participants did not reveal significant differences; most exited participants had stage III/IV PDAC and have died (Table S1).

### 3.4. Survey Completion

Over an 18-month period, 189 baseline surveys were completed (62% completion rate). Of the 189 participants who completed the baseline survey, 164 reached follow-up 1 and 76 completed that survey (46% completion rate). To increase completion rates, participants receive automated email and/or phone call reminders from site coordinators.

### 3.5. Computed Tomography (CT) Scan Acquisition

Baseline CT images from 231 participants (178 NHW, 15 AA, 38 H/L) have been uploaded to our central imaging repository (Figure 3). Most scans are ‘CT Abdomen Pelvis’ (*n* = 73, 32%). ‘CT Abdomen’ and ‘CT Thorax Abdomen Pelvis’ account for 26% of scans (*n* = 60), followed by ‘Pancreas Protocol CT’ scans (*n* = 30, 13%) and other types. CT scans from follow-up time points 1 and 2 have been received for 20 and 2 participants, respectively.

### 3.6. Biospecimen Collection

Blood samples have been obtained at baseline for 264 participants (198 NHW, 23 AA, 43 H/L) and at follow-up timepoints 1 and 2 for 77 and 27 participants, respectively (Figure 3). Tissue samples have been collected from 159 of 175 surgical cases, with 119 matched PT-NP pairs, 152 AD-O, and 149 MU samples collected. Most pancreatic tumor samples (*n* = 114, 91.9%) were collected prior to any treatment, while ten samples were collected post-treatment. Of the 159 participants with available tissue samples, most had a diagnosis of PDAC (52%, *n* = 82) followed by IPMNs (15%, *n* = 24) and PNETs (12%, *n* = 19). 

## 4. Discussion

We describe the establishment of the first state-wide biobank dedicated to minimizing disparities and personalizing care for individuals affected by PaCa. Through this multiple stakeholder-led initiative, we developed and implemented robust SOPs to collect, process, and store blood and tissues uniformly to ensure quality specimens for downstream analyses. Moreover, we developed standardized methods for the collection of data and images with which to annotate the biospecimens. By integrating these resources, we hope to investigate biological processes that may underlie disparities and poor outcomes and develop targeted interventions that may improve outcomes and equity. In line with prior studies of PaCa disparities [4,6,7,8,9,10,11,17,35], the AA cases enrolled in our cohort to date were diagnosed younger (mean = 64 years) and have a higher BMI than NHW. In contrast to retrospective reviews of cancer registry data from Texas [8] and California [10], we are observing a higher proportion of AA and H/L females affected by PaCa in our cohort. 

It is well documented that recruitment and enrollment of underserved populations into biobanks and clinical trials is challenging due to lack of trust, privacy concerns, language barriers, aversion to blood draws, transportation issues and institutional barriers [36,37,38,39,40,41,42]. The FPC has worked diligently to address these barriers through incorporation of known facilitators, including use of Spanish-translated written materials, combining study visits and blood draws with SOC appointments, and enlisting promotion of the study by engaged providers trusted by potential participants [41,43]. To address institutional barriers, MCC has worked closely with participating sites to meet with pathologists, laboratory technicians, regulatory specialists, and business offices to obtain study buy-in, ensure a coordinator is available for study visits and data entry, and that study-related supplies (including iPads) are provided. This institutional support has been essential to ensuring the study is accessible to populations seen at community hospitals and/or those without research infrastructure. 

**Figure 4:**
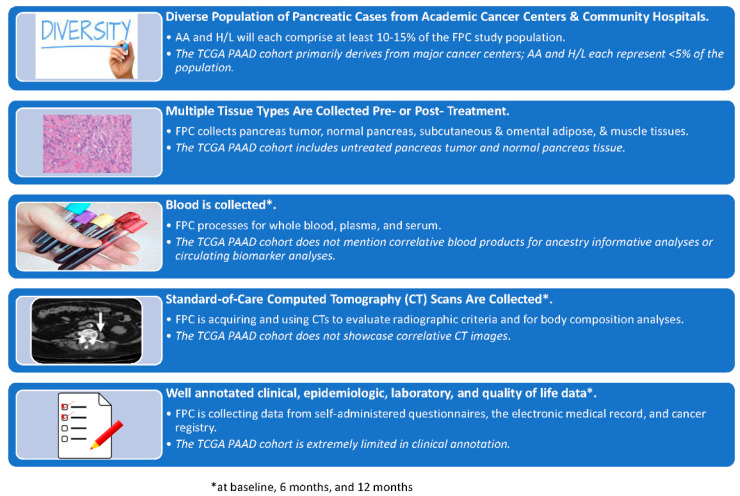
Resources Ascertained through the Florida Pancreas Collaborative (FPC) versus the Pancreatic Ductal Adenocarcinoma (PAAD) Cohort included in The Cancer Genome Atlas (TCGA) Pan-Cancer Analyses. This comparison reveals that the FPC: (a) includes participants recruited from the community and has a higher proportion of underserved minority populations, (b) collects tissue types (untreated and treated) in addition to pancreas tumors and normal pancreas, and has (c) correlative blood, (d) CT scans, and (e) well-annotated data corresponding to each participant. Note: The PAAD cohort being referred to was published by Liu et al. [48].

The 30 AA and 47 H/L individuals enrolled to date account for 9.8% and 15.4% of total participants recruited, respectively, representing percentages higher than those reported in existing pancreas biobanks [22,23] and molecular studies of pancreatic tumors from TCGA and other initiatives [44,45,46,47,48]. A comparison of the FPC resource with the PDAC TCGA cohort [48] underscores how our collaborative is filling important gaps in PaCa disparities research by: including a greater representation of minority groups and collecting untreated and treated tissue types in addition to pancreas tumor tissue along with CT scans, blood, and a comprehensive set of clinical, epidemiologic, laboratory, and quality of life variables (Figure 4). Importantly, compared to other biospecimen donation studies [36,49,50], the willingness of eligible AA and H/L patients to participate has been remarkably high at 100% and 94%, respectively. Thus, despite what may appear to be relatively small sample sizes, the FPC has experienced success enrolling underserved populations at sites selected based on state data [25]. With continued recruitment, we expect numbers of AA and H/L cases to increase, particularly with the activation of our two remaining sites which see a high volume of AA and H/L patients. Furthermore, we expect to accelerate enrollment as remote recruitment efforts are increasingly adopted by sites in response to the COVID-19 pandemic. The FPC recognizes challenges associated with accessing underserved populations throughout the state who may be pursuing care at hospitals and facilities that do not facilitate guideline-concordant treatment. We are pursuing opportunities to engage and educate providers to bridge this gap and ensure appropriate treatment is provided.

In terms of immediate plans, we are working to advance cancer cachexia research using data elements collected via the study questionnaire and health screen, laboratory values from the EMR, and quantitative CT imaging metrics (visceral adiposity, skeletal muscle index, and psoas muscle index), and serum biomarkers (such as cytokines and adipokines). We will also be performing genotyping for ancestry informative markers to validate self-reported ancestry and plan to conduct molecular profiling of tumor tissue. Finally, based on the promise of pre-clinical models in translational efforts, we plan to leverage the pancreatic tissue collected and preserved through this effort for applications such as generation of patient-derived organoids [51]. Thus, the FPC will become an enduring resource for the biomedical and disparities community. 

Investigators in and outside the FPC may request to collaborate and utilize these resources to evaluate new research hypotheses after a series of analyses are conducted and published by the FPC. A written proposal would be submitted and reviewed by the FPC Biobank Utilization Committee with decisions made based upon peer-review of scientific merit, specimen availability, experience of the requesting investigator(s), and adequate resources to conduct proposed methods. Samples and/or data would be released upon committee approval once the requestors secure regulatory approvals, conflicts of interest disclosures are reviewed, and data use and material transfer agreements are established. Intellectual property issues would be agreed upon in advance with results from the new research findings incorporated into the biobank. In this manner, the FPC data repository continues to evolve, generating new correlations and opportunities to evaluate hypotheses.

## 5. Conclusions

In summary, multidisciplinary, multi-institutional collaborations in partnership with community stakeholders are key to successfully addressing PaCa disparities. Institutions must commit to fostering health disparities research and to eliminating racism as a root of inequity. It is our intent that the infrastructure-building described here can serve as a model for other teams who wish to develop similar resources applicable to their disease sites. 

## Figures and Tables

**Table 1:** Summary of Florida Pancreas Collaborative biobank contents and time-points for collection.

Biobank Contents	Time-Point		
	Baseline	6 months	12 months
Health Screen	√	√	√
Study Questionnaire	√	√	√
Clinical, laboratory, imaging, and pathologic data abstracted from the medical record and/or requested from the Florida Cancer Data System	√	√	√
Blood processed for plasma and serum (and DNA for ancestry analysis in the future)	√	√	√
Tissue from surgery (or biopsy): pancreas tumor (PT), normal pancreas (NP), adipose-subcutaneous (AD-S), adipose-omental (AD-O), and muscle (MU)	√	a	a
Computed tomography (CT) images	√	√	√

a Tissue to be collected for research if a procedure is being performed as part of clinical care.

**Table 2:** Data elements solicited in the Florida Pancreas Collaborative Baseline Study Questionnaire or Case Report Forms (CRF).

Baseline Questionnaire		Case Report Forms	
Section	Information Requested	CRF/Module	Information Requested
Demographics	Age, gender identity, race, ethnicity, marital status, education	Chief Complaints and Comorbidities	Detailed list of presenting symptoms and comorbidities
	Insurance status, occcupational history		Performance status-Eastern Cooperative Oncology Group (ECOG)
Personal History of Cancer	Cancer type(s), age(s) at diagnosis, treatment(s) received	Anthropometrics and Lab Values	Height, weight, body mass index (BMI) and weight-to-hip-ratio (WHR)
and other medical conditions	Condition name(s), age(s) at diagnosis, treatment(s) received, cancer screening history		Serum CRP, bilirubin, albumin, CEA and CA 19-9 levels. Pancreatic cyst fluid: amylase, CEA and CA 19-9 levels
Risk factors	Height, weight, dietary history, physical activity	Radiologic Reporting	Type(s) and date(s) of imaging performed (e.g., MRI, CT or EUS). Pancreatic parenchymal phase (appearance, size and location). Pancreatic duct narrowing dilatation, termination
	Menstrual and reproductive history (females only), alcohol consumption, tobacco and medical marijuana use, sleep habits		Evaluation of arterial, venous and extrapancreatic contact. Impression: tumor size and location
	Medication use (aspirin, statins, metformin), chemical exposures		Metastases-location
Family history of cancer	Diagnosis	Staging	Clinical staging
and other medical conditions	Family member’s relation to proband	Radiology Body Composition Analysis	Abdominal/visceral adiposity
	Age at diagnosis, genetic testing results		Psoas index, skeletal muscle index
Social support and quality of life	Cancer-specific functional scales	Diagnosis and Treatment Recommendations	Diagnosis
	Pancreatic cancer related symptoms		Surgical recommendation
	Patient’s perspective on optimism vs pessimism		* Types of neo-adjuvant therapy, including drug(s) and dose(s) Neo-adjuvant therapy start and end date
			* Types of adjuvant therapy, including drug(s) and doses Adjuvant therapy start and end date
		Surgery	American Society of Anesthesiologists (ASA) class
			** Type of procedures performed (ie whipple, distal pancreatectomy), lymph nodes (total and number positive)
			Size and location of lesion, post-op diagnosis
			Drains placed in operating room (OR) (total and type), stent placement
			Estimated blood loss, pancreatic gland texture
			Vascular resection and type of reconstruction, feeding tube placement
		Pathology	Histology/Behavior (ICD-0-3), grade, size
			Tumor (T) nodes (N) and metastases (M) stage Pancreatic, biliary and SMA margin status
			Lymph node involvement (total examined, number positive) Grades of IPMN and PanIN involved, if applicable
		Post-op Course and Complications	Complication type(s)
			Total parenteral nutrition and tube feed status
			Leaks present (non-pancreas, anastomotic, pancreatic fistula). Detailed list of conditions presented during post-op
			Length of intensive care unit and hospital stay Post-op death status
			Diet on discharge, reasons for readmission
		Follow-up	Date of last patient contact, vital status, recurrence status (date, treatment type)
			Overall, disease free and disease specific survival (in months)

Notes: * Chemotherapy, radiation, immunotherapy, hormone or targeted therapy; ** Surgery, diagnostic staging laparoscopy, intra-operative ultrasound, and frozen section. Abbreviations: CA-19-9 = Cancer antigen 19-9; CRP = C-reactive protein; WBC = White blood cells; CEA = Carcinoembryonic antigen; MRI = magnetic resonance imaging; CT = computed tomography; EUS = endoscopic ultrasound; SMA = Superior mesenteric artery; IPMN = intraductal papillary mucinous neoplasm; PanIN = pancreatic intraepithelial neoplasia.

**Table 3:** Validated instruments incorporated into the FPC health screen or comprehensive questionnaire.

Survey Name	Abbreviation	Where Administered	Purpose of Survey
Edmonton Symptom Assessment Scale	ESAS	Health Screen	To assess nine commonly observed symptoms in cancer patients i.e. pain, tiredness, nausea, depression, anxiety, drowsiness, appetite, wellbeing and shortness of breath and determine the clinical profile of the symptoms over time.
Patient Generated - Subjective Global Assessment Short form	PG-SGA	Health Screen	PG-SGA consists of four main sections, ie. Weight, Food Intake, Symptoms and Activities that helps to determine the functional status of the patient.
Pittsburgh Sleep Quality Index	PSQI	Questionnaire	In PSQI, using the 19 individual items, 7 "component scores" are generated, assessing sleep quality, sleep latency, duration, habitual sleep efficiency, sleep disturbances, use of sleep medications and daytime dysfunction. Information is collected for the past one month.
Cancer Patient Tobacco Use Questionnaire	C-TUQ	Questionnaire	NCI AACR Cancer Patient Tobacco Use Assessment Task Force developed and validated the C-TUQ. The survey collects information on smoking status, smoking history and status relative to cancer diagnosis and treatment, use of tobacco products and secondhand smoke exposure and cessation.
European Organization for Research and Treatment of Cancer – Quality of Life of Cancer Patients	EORTC QLQ-C30	Questionnaire	QLQ C30 is a cancer-specific quality of life questionnaire consisting of five functional scales, three symptom scales, an overall health status and commonly reported symptoms by cancer patients and perceived financial effect of the disease.
European Organization for Research and Treatment of Cancer – Pancreatic Cancer (in phase III of testing)	EORTC PAN26	Questionnaire	QLQ-PAN26 consists of 26 four level likert scale questions focussing on pancreatic pain scale referring to abdominal discomfort, back pain, pain during night and discomfort in certain positions.
Enhancing Recovery in Coronary Heart Disease Social Support Inventory	ENRICHD-ESSI	Questionnaire	ESSI is a seven item survey measuring the range of social support in the patients life using a Likert scale for the first 6 questions.
Life Orientation Test – Revised	LOTR	Questionnaire	LOT-R includes 10 questions and helps in determining the individual differences in generalized optimism versus pessimism. The revised version also adds more details on expections for the future.
Stress and Adversity Inventory	STRAIN	Questionnaire	A stress assessment tool available online and evaluating the patient’s exposure to acute and chronic stress throughout their lifetime.
Dietary Screener Questionnaire	DSQ	Questionnaire	DSQ includes dietary factors that are of interest in cancer and heart disease and collects dietary intake over the past month.

**Table 4:** Baseline demographic and clinical characteristics of Florida Pancreas Collaborative study participants, by race/ethnicity.

Variable	All Participants (*N* = 305)	African American (*N* = 30)	Hispanic/Latinx (*N* = 47)	Non-Hispanic White (*N* = 228)	*p*-Value
Age (years), mean (± SD)	68 (10.6)	64 (12.1)	63 (12.8)	70 (9.4)	0.0001
Gender, *n* (%)					
Female	161 (52.8)	17 (56.7)	30 (63.8)	114 (50%)	0.2028
Male	144 (47.2)	13 (43.3)	17 (36.2)	114 (50%)	
Education level ^†^, *n* (%)					
High school or GED	46 (31.1)	6 (54.5)	6 (42.9)	34 (27.6)	0.1599
College	65 (43.9)	4 (36.4)	7 (50.0)	54 (43.9)	
Postgraduate	37 (25.0)	1 (9.1)	1 (7.1)	35 (28.5)	
Data not yet available ^Φ^	157	19	33	105	
Income Level ^†^, *n* (%)					
Below $40k	38 (26.0)	6 (54.5)	4 (30.8)	28 (22.9)	0.2049
$40k–100k	42 (28.8)	2 (18.2)	3 (23.1)	37 (30.3)	
100k and above	34 (23.3)	1 (9.1)	1 (7.7)	32 (26.3)	
Information not provided by Participant	32 (21.9)	2 (18.2)	5 (38.5)	25 (20.5)	
Data not yet available ^Φ^	159	19	34	106	
Health Insurance ^†^, *n* (%)					
Insured	143 (97.9)	10 (90.9)	13 (100.0)	120 (98.4)	0.2301
Uninsured	3 (2.1)	1 (9.1)	0 (0.0)	2 (1.6)	
Data not yet available ^Φ^	159	19	34	106	
Marital Status ^†^, *n* (%)					
Not married	38 (26.0)	5 (45.5)	2 (15.4)	31 (25.4)	0.0376
Married	107 (73.2)	5 (45.5)	11 (84.6)	91 (74.6)	
Information not provided by Participant	1 (0.8)	1 (9.0)	0 (0.0)	0 (0.0)	
Data not yet available	159	19	34	106	
Family History of Pancreatic Cancer ^†^, *n* (%)					
No	87 (67.4)	8 (72.7)	6 (66.7)	73 (67.0)	0.8005
Yes	16 (12.4)	0 (0.0)	1 (11.1)	15 (13.8)	
Participant does not know	26 (20.2)	3 (27.3)	2 (22.2)	21 (19.2)	
Data not yet available ^Φ^	176	19	38	119	
Distress ^‡^, *n* (%)					
No	36 (12.4)	3 (11.1)	7 (14.9)	26 (12.0)	0.8309
Yes	255 (87.6)	24 (88.9)	40 (85.1)	191 (88.0)	
Data not yet available ^Φ^	14	3	0	11	
Depression ^‡^, *n* (%)					
No	189 (64.9)	20 (74.1)	33 (70.2)	136 (62.7)	0.3579
Mild depression	43 (14.8)	4 (14.8)	3 (6.4)	36 (16.6)	
Moderate depression	40 (13.8)	1 (3.7)	7 (14.9)	32 (14.7)	
Severe depression	19 (6.5)	2 (7.4)	4 (6.4)	13 (6.0)	
Data not yet available ^Φ^	14	3	0	11	
Smoking status ^†‡^, *n* (%)					
No	129 (44.3)	14 (50)	32 (68.1)	83 (38.4)	0.0055
Former smoker	127 (43.6)	10 (35.7)	12 (25.5)	105 (48.6)	
Current smoker	35 (12.1)	4 (14.3)	3 (6.4)	28 (13.0)	
Data not yet available ^Φ^	14	2	0	12	
Marijuana status ^†^, *n* (%)					
No	97 (71.9)	5 (50)	10 (90.9)	82 (71.9)	0.1662
Former user	25 (18.5)	3 (30)	0 (0.0)	22 (19.3)	
Current user	13 (9.6)	2 (20)	1 (9.1)	10 (8.8)	
Data not yet available ^Φ^	170	20	36	114	
Abdominal Pain ^¶^, *n* (%)					
No	78 (38.8)	12 (46.2)	11 (31.4)	55 (39.3)	0.0948
Yes	100 (49.8)	13 (50.0)	23 (65.7)	64 (45.7)	
Information unavailable in EMR	23 (11.4)	1 (3.8)	1 (2.9)	21 (15.0)	
Data not yet available ^Φ^	104	4	12	88	
Fatigue ^¶^, *n* (%)					
No	148 (52.5)	15 (53.6)	20 (45.5)	113 (53.8)	0.0975
Yes	98 (34.7)	12 (42.8)	21 (47.7)	65 (31.0)	
Information unavailable in EMR	36 (12.8)	1 (3.6)	3 (6.8)	32 (15.2)	
Data not yet available ^Φ^	23	2	3	18	
GI Bleeding ^¶^, *n* (%)					
No	217 (77.0)	23 (82.1)	39 (88.6)	155 (73.8)	0.0346
Yes	7 (2.5)	2 (7.1)	1 (2.3)	4 (1.9)	
Information unavailable in EMR	58 (20.5)	3 (10.8)	4 (9.1)	51 (24.3)	
Data not yet available ^Φ^	23	2	3	18	
Jaundice ^¶^, *n* (%)					
No	178 (62.9)	22 (78.6)	25 (55.6)	131 (62.4)	0.0129
Yes	66 (23.3)	4 (14.3)	18 (40.0)	44 (21.0)	
Information unavailable in EMR	39 (13.8)	2 (7.1)	2 (4.4)	35 (16.6)	
Data not yet available ^Φ^	22	2	2	18	
Weight Loss More than 5% ^¶^, *n* (%)					
No	133 (47.4)	22 (78.6)	25 (55.6)	131 (62.4)	0.0972
Yes	115 (40.9)	4 (14.3)	18 (40.0)	44 (21.0)	
Information unavailable in EMR	33 (11.7)	2 (7.1)	2 (4.4)	35 (16.6)	
Data not yet available ^Φ^	24	2	2	18	
Charlsons Comorbidity Index, *n* (%)					
0	164 (57.7)	16 (57.1)	27 (60.0)	121 (57.3)	0.6516
≤2	101 (35.6)	11 (39.3)	13 (28.9)	77 (36.5)	
≥3	19 (6.7)	1 (3.6)	5 (11.1)	13 (6.2)	
Data not yet available ^Φ^	21	2	2	17	
Personal History of Diabetes ^†¶^, *n* (%)					
No	195 (68.4)	21 (75)	27 (60)	147 (69.3)	0.3464
Yes	90 (31.6)	7 (25)	18 (40)	65 (30.7)	
Data not yet available ^Φ^	20	2	2	16	
Personal History of Pancreatitis ^†¶^, *n* (%)					
No	180 (79.6)	23 (85.2)	32 (86.5)	125 (77.2)	0.3745
Yes	46 (20.4)	4 (14.8)	5 (13.5)	37 (22.8)	
Data not yet available ^Φ^	79	3	10	66	
Cachexia ^‡¶^, *n* (%)					
refractory cachexia	1 (3.8)	0 (0.0)	4 (8.9)	6 (3.1)	0.3098
Cachexia	76 (29.1)	4 (18.2)	16 (35.6)	56 (28.9)	
pre-cachexia	26 (10.0)	4 (18.2)	2 (4.4)	20 (10.3)	
non cachectic	149 (57.1)	14 (63.6)	23 (51.1)	112 (57.7)	
Missing	44	8	2	34	
Body Mass Index (kg/m^2^) ^¶^, *n*, mean (SD)	281, 27 (5.5)	26, 28 (5.2)	44, 25 (5.0)	211, 27 (5.5)	0.0348
Waist Circumference ^¶^, n, mean (SD)	231, 40 (12.7)	17, 39 (6.2)	37, 38 (13.5)	177, 40 (13.0)	0.6594
Histology ^¶^, *n* (%)					
Pancreatic ductal adenocarcinoma (PDAC)	183 (61.4)	16 (57.2)	36 (78.3)	131 (58.5)	0.0200
Pancreatic neuroendocrine tumor (PNET)	35 (11.7)	7 (25.0)	3 (6.5)	25 (11.1)	
Intraductal papillary mucinous neoplasm (IPMN)	35 (11.7)	1 (3.6)	2 (4.3)	32 (14.3)	
Mucinous cystic neoplasm (MCN)	6 (2.0)	2 (7.1)	0 (0.0)	4 (1.8)	
Other ^§^	39 (13.1)	2 (7.1)	5 (10.9)	32 (14.3)	
Data not yet available ^Φ^	7	2	1	4	
Surgical Resection Attempted ^¶^, *n* (%)					
No	146 (47.9)	17 (56.7)	26 (55.3)	103 (45.2)	0.2673
Yes	159 (52.1)	13 (43.3)	21 (44.7)	125 (54.8)	
Location of Tumor ^¶^, *n* (%)					
Body	16 (14.0)	2 (33.3)	1 (4.8)	13 (13.3)	0.3351
Diffuse	17 (14.9)	2 (33.3)	3 (14.3)	12 (12.4)	
Head	65 (57.0)	1 (16.7)	13 (61.8)	51 (52.6)	
Tail	13 (11.4)	0 (0.0)	1 (4.8)	12 (12.4)	
Other	13 (11.4)	1 (16.7)	3 (14.3)	9 (9.3)	
Data not yet available ^Φ^	181	24	26	131	
Stage ^¶^, *n* (%)					
Stage 0	22 (14.1)	1 (20.0)	1 (4.5)	20 (16.3)	0.6547
Stage I/II	87 (55.8)	3 (60.0)	14 (63.7)	70 (56.9)	
Stage III/IV	41 (26.3)	1 (20.0)	7 (31.8)	33 (26.8)	
Data not yet available ^Φ^	155	25	25	105	
Grade Exocrine Pancreatic Tumors ^¶¥^, *n* (%)					
Well differentiated	7 (8.0)	0 (0.0)	3 (15.8)	4 (6.1)	0.1719
Moderately differentiated	29 (32.9)	0 (0.0)	9 (47.4)	20 (30.3)	
Poorly differentiated	21 (23.9)	1 (33.3)	4 (21.0)	16 (24.2)	
Grade undetermined	31 (35.2)	2 (66.7)	3 (15.8)	26 (39.4)	
Data not yet available ^Φ^	136	16	19	101	
Grade IPMN ^¶^, *n* (%)					
Low grade	12 (34.3)	0 (0.0)	1 (50.0)	11 (34.4)	1.0000
Borderline	2 (5.7)	0 (0.0)	0 (0.0)	2 (6.3)	
Carcinoma-in-situ	5 (14.3)	0 (0.0)	0 (0.0)	5 (15.6)	
Invasive carcinoma	1 (2.9)	0 (0.0)	0 (0.0)	1 (3.1)	
Unknown grade	15 (42.9)	1 (100.0)	1 (50.0)	13 (40.6)	
Positive Lymph Nodes ^¶^, *n* (%)					
No	110 (94.8)	5 (100.0)	17 (89.5)	88 (95.7)	0.6800
Yes	6 (5.2)	0 (0.0)	2 (10.5)	4 (4.3)	
Data not yet available ^Φ^	189	25	28	136	

† Data available from partially-or fully-completed baseline questionnaires at time of analysis. ^Φ^ Data not yet available. Additional data will be included in future analyses when entered into the DatStat system. ‡ Data available from the health screen questionnaire at time of analysis. ¶ Data available from case report forms (CRFs) at time of analysis. § The ‘Other’ category includes benign and malignant tumors of pancreatic, liver and bile duct, renal, adrenal gland, lymph node, and unclassified origin. ¥ The grade for exocrine tumors has been restricted to PDAC, IPMN, and MCN (n = 224). Categorization of cachexia was performed using methods described by Vigano et al. [33]. Categorization of depression was performed using methods described by Oldenmenger et al. [34].

## Data Availability

The data presented in this study are available on request from the corresponding author.

## Supplementary Materials

The following are available online at https://www.mdpi.com/2072-6694/13/4/809/s1, Figure S1: Workflow of Main Study-related Tasks Accomplished with the Assistance of our Online Data Management/Engagement Platform, Figure S2: Workflow and Modules Represented in the Electronic-Consent (e-consent) Process, Figure S3: Examples of Data Management and Tracking Screens, Figure S4: Florida Pancreas Collaborative Health Screen Instrument, Figure S5: Example of a Health Screen Report Obtained at Baseline, Figure S6: Florida Pancreas Collaborative Data Repository (FPCDR) Infrastructure, Figure S7: Total number of participants enrolled in the Florida Pancreas Collaborative, by study site and race/ethnicity, Table S1: Sensitivity analysis comparing demographic and clinical characteristics by follow-up status.

## References

[1] American Cancer Society (2020). Cancer Facts and Figures 2020.

[2] Rahib L., Smith B.D., Aizenberg R., Rosenzweig A.B., Fleshman J.M., Matrisian L.M. (2014). Projecting cancer incidence and deaths to 2030: The unexpected burden of thyroid, liver, and pancreas cancers in the United States. Cancer Res..

[3] Howlader N., Noone A.M., Krapcho M., Miller D., Bishop K., Altekruse S.F., Kosary C.L., Yu M., Ruhl J., Tatalovich Z. (2016). SEER Cancer Statistics Review, 1975–2013.

[4] Abraham A., Al-Refaie W.B., Parsons H.M., Dudeja V., Vickers S.M., Habermann E.B. (2013). Disparities in pancreas cancer care. Ann. Surg. Oncol..

[5] Chang K.J., Parasher G., Christie C., Largent J., Anton-Culver H. (2005). Risk of pancreatic adenocarcinoma: Disparity between African Americans and other race/ethnic groups. Cancer.

[6] Riall T.S., Townsend C.M., Kuo Y.F., Freeman J.L., Goodwin J.S. (2010). Dissecting racial disparities in the treatment of patients with locoregional pancreatic cancer: A 2-step process. Cancer.

[7] Singal V., Singal A.K., Kuo Y.F. (2012). Racial disparities in treatment for pancreatic cancer and impact on survival: A population-based analysis. J. Cancer Res. Clin. Oncol..

[8] Wray C.J., Castro-Echeverry E., Silberfein E.J., Ko T.C., Kao L.S. (2012). A multi-institutional study of pancreatic cancer in Harris County, Texas: Race predicts treatment and survival. Ann. Surg. Oncol..

[9] Murphy M.M., Simons J.P., Hill J.S., McDade T.P., Chau Ng S., Whalen G.F., Shah S.A., Harrison L.H., Tseng J.F. (2009). Pancreatic resection: A key component to reducing racial disparities in pancreatic adenocarcinoma. Cancer.

[10] Zell J.A., Rhee J.M., Ziogas A., Lipkin S.M., Anton-Culver H. (2007). Race, socioeconomic status, treatment, and survival time among pancreatic cancer cases in California. Cancer Epidemiol. Biomark. Prev..

[11] Murphy M.M., Simons J.P., Ng S.C., McDade T.P., Smith J.K., Shah S.A., Zhou Z., Earle C.C., Tseng J.F. (2009). Racial differences in cancer specialist consultation, treatment, and outcomes for locoregional pancreatic adenocarcinoma. Ann. Surg. Oncol..

[12] DeSantis C.E., Siegel R.L., Sauer A.G., Miller K.D., Fedewa S.A., Alcaraz K.I., Jemal A. (2016). Cancer statistics for African Americans, 2016: Progress and opportunities in reducing racial disparities. CA Cancer J. Clin..

[13] American Cancer Society (2013). Cancer Facts and Figures for African Americans 2013-2014.

[14] American Cancer Society (2012). Cancer Facts and Figures for Hispanics/Latinos 2012-2014.

[15] F.C.D.S. http://fcds.med.miami.edu/.

[16] Permuth J.B., Clark Daly A., Jeong D., Choi J.W., Cameron M.E., Chen D.T., Teer J.K., Barnett T.E., Li J., Powers B.D. (2019). Racial and ethnic disparities in a state-wide registry of patients with pancreatic cancer and an exploratory investigation of cancer cachexia as a contributor to observed inequities. Cancer Med..

[17] Arnold L.D., Patel A.V., Yan Y., Jacobs E.J., Thun M.J., Calle E.E., Colditz G.A. (2009). Are racial disparities in pancreatic cancer explained by smoking and overweight/obesity?. Cancer Epidemiol. Biomark. Prev..

[18] Meguid R.A., Ahuja N., Chang D.C. (2008). What constitutes a “high-volume” hospital for pancreatic resection?. J. Am. Coll. Surg..

[19] Noel M., Fiscella K. (2019). Disparities in Pancreatic Cancer Treatment and Outcomes. Health Equity.

[20] Silverman D.T., Hoover R.N., Brown L.M., Swanson G.M., Schiffman M., Greenberg R.S., Hayes R.B., Lillemoe K.D., Schoenberg J.B., Schwartz A.G. (2003). Why do Black Americans have a higher risk of pancreatic cancer than White Americans?. Epidemiology.

[21] Boyd R., Lindo E.G., Weeks L.D., McLemore M.R. (2020). On Racism: A New Standard For Publishing On Racial Health Inequities. Health Aff. Blog.

[22] Yadav D., Park W.G., Fogel E.L., Li L., Chari S.T., Feng Z., Fisher W.E., Forsmark C.E., Jeon C.Y., Habtezion A. (2018). PROspective Evaluation of Chronic Pancreatitis for EpidEmiologic and Translational StuDies: Rationale and Study Design for PROCEED From the Consortium for the Study of Chronic Pancreatitis, Diabetes, and Pancreatic Cancer. Pancreas.

[23] Hwang R.F., Wang H., Lara A., Gomez H., Chang T., Sieffert N., Moon Y., Ram S., Zimmerman S., Lee J.H. (2008). Development of an integrated biospecimen bank and multidisciplinary clinical database for pancreatic cancer. Ann. Surg. Oncol..

[24] Permuth J.B., Trevino J., Merchant N., Malafa M. (2016). Partnering to advance early detection and prevention efforts for pancreatic cancer: The Florida Pancreas Collaborative. Future Oncol..

[25] Agency for Health Care Administration. http://www.fchc.state.fl.us.

[26] Permuth-Wey J., Borenstein A.R. (2009). Financial Remuneration for Clinical and Behavioral Research Participation: Ethical and Practical Considerations. Ann. Epidemiol..

[27] Lang R., Kelkar V.A., Byrd J.R., Edwards C.L., Pericak-Vance M., Byrd G.S. (2013). African American participation in health-related research studies: Indicators for effective recruitment. J. Public Health Manag. Pract..

[28] Perez D.F., Nie J.X., Ardern C.I., Radhu N., Ritvo P. (2013). Impact of participant incentives and direct and snowball sampling on survey response rate in an ethnically diverse community: Results from a pilot study of physical activity and the built environment. J. Immigr. Minor. Health.

[29] Schnieders T., Danner D.D., McGuire C., Reynolds F., Abner E. (2013). Incentives and barriers to research participation and brain donation among African Americans. Am. J. Alzheimers Dis. Demen..

[30] Clark K., Vendt B., Smith K., Freymann J., Kirby J., Koppel P., Moore S., Phillips S., Maffitt D., Pringle M. (2013). The Cancer Imaging Archive (TCIA): Maintaining and operating a public information repository. J. Digit. Imaging..

[31] Al-Hawary M.M., Francis I.R., Chari S.T., Fishman E.K., Hough D.M., Lu D.S., Macari M., Megibow A.J., Miller F.H., Mortele K.J. (2014). Pancreatic ductal adenocarcinoma radiology reporting template: Consensus statement of the society of abdominal radiology and the american pancreatic association. Gastroenterology.

[32] Slavich G.M., Shields G.S. (2018). Assessing Lifetime Stress Exposure Using the Stress and Adversity Inventory for Adults (Adult STRAIN): An Overview and Initial Validation. Psychosom. Med..

[33] Vigano A., Del Fabbro E., Bruera E., Borod M. (2012). The cachexia clinic: From staging to managing nutritional and functional problems in advanced cancer patients. Crit. Rev. Oncog..

[34] Oldenmenger W.H., de Raff P.J., de Klerk C., van der Rijt C.C.D. (2013). Cut points on 0-10 numeric rating scales for symptoms incuded in the Edmonton Symptom Assessment Scale in cancer patients: A systematic review. J. Pain Symptom Manag..

[35] Chang J.I., Huang B.Z., Wu B.U. (2018). Impact of Integrated Health Care Delivery on Racial and Ethnic Disparities in Pancreatic Cancer. Pancreas.

[36] Luque J.S., Quinn G.P., Montel-Ishino F.A., Arevalo M., Bynum S.A., Noel-Thomas S., Wells K.J., Gwede C.K., Meade C.D. (2012). Formative research on perceptions of biobanking: What community members think. J. Cancer Educ..

[37] Schmotzer G.L. (2012). Barriers and facilitators to participation of minorities in clinical trials. Ethn. Dis..

[38] Sheppard V.B., Mays D., LaVeist T., Tercyak K.P. (2013). Medical mistrust influences black women’s level of engagement in BRCA 1/2 genetic counseling and testing. J. Natl. Med. Assoc..

[39] Halbert C.H., McDonald J., Vadaparampil S., Rice L., Jefferson M. (2016). Conducting Precision Medicine Research with African Americans. PLoS ONE.

[40] Reddy A., Amarnani A., Chen M., Dynes S., Flores B., Moshchinsky A., Lee Y.J., Kurbatov V., Shapira I., Vignesh S. (2020). Privacy Concerns About Personal Health Information and Fear of Unintended Use of Biospecimens Impact Donations by African American Patients. J. Cancer Educ..

[41] Davis T.C., Arnold C.L., Mills G., Miele L. (2019). A Qualitative Study Exploring Barriers and Facilitators of Enrolling Underrepresented Populations in Clinical Trials and Biobanking. Front. Cell Dev. Biol..

[42] Joseph G., Dohan D. (2009). Recruiting minorities where they receive care: Institutional barriers to cancer clinical trials recruitment in a safety-net hospital. Contemp. Clin. Trials.

[43] Cook E.D., Yeager K.A., Cecchini R.S., Boparai J., Brown C.L., Duncan M., Cronin W.M., Paskett E.D. (2018). Recruitment practices for U.S. minority and underserved populations in NRG oncology: Results of an online survey. Contemp. Clin. Trials Commun..

[44] Bailey P., Chang D.K., Nones K., Johns A.L., Patch A.M., Gingras M.C., Miller D.K., Christ A.N., Bruxner T.J., Quinn M.C. (2016). Genomic analyses identify molecular subtypes of pancreatic cancer. Nature.

[45] Collisson E.A., Sadanandam A., Olson P., Gibb W.J., Truitt M., Gu S., Cooc J., Weinkle J., Kim G.E., Jakkula L. (2011). Subtypes of pancreatic ductal adenocarcinoma and their differing responses to therapy. Nat. Med..

[46] Moffitt R.A., Marayati R., Flate E.L., Volmar K.E., Loeza S.G., Hoadley K.A., Rashid N.U., Williams L.A., Eaton S.C., Chung A.H. (2015). Virtual microdissection identifies distinct tumor-and stroma-specific subtypes of pancreatic ductal adenocarcinoma. Nat. Genet..

[47] Raphael B.J., Hruban R.H., Aguirre A.J., Moffitt R.A., Yeh J.J., Stewart C. (2017). Integrated Genomic Characterization of Pancreatic Ductal Adenocarcinoma. Cancer Cell.

[48] Liu J., Lichtenberg T., Hoadley K.A., Poisson L.M., Lazar A.J., Cherniack A.D., Kovatich A.J., Benz C.C., Levine D.A., Lee A.V. (2018). An Integrated TCGA Pan-Cancer Clinical Data Resource to Drive High-Quality Survival Outcome Analytics. Cell.

[49] Kiviniemi M.T., Saad-Harfouche F.G., Ciupak G.L., Davis W., Moysich K., Hargrave N.C., Ambrosone C.B., Walker C., Erwin D.O. (2013). Pilot intervention outcomes of an educational program for biospecimen research participation. J. Cancer Educ..

[50] Adams-Campbell L.L., Dash C., Palmer J.R., Wiedemeier M.V., Russell C.W., Rosenberg L., Cozier Y.C. (2016). Predictors of biospecimen donation in the Black Women’s Health Study. Cancer Causes Control.

[51] Beato F., Reverón D., Dezsi K.B., Ortiz A., Johnson J.O., Chen D.T., Ali K., Yoder S.J., Jeong D., Malafa M. (2020). Establishing a living biobank of patient-derived organoids of intraductal papillary mucinous neoplasms of the pancreas. Lab. Investig..

