# Supplementary material for: The Florida Pancreas Collaborative Next-Generation Biobank: Infrastructure to Reduce Disparities and Improve Survival for a Diverse Cohort of Patients with Pancreatic Cancer
Source: Cancers (Basel). 2021 Feb 15;13(4):809. doi: 10.3390/cancers13040809 (PMC7919015; doi:10.3390/cancers13040809)
Supplement: Supplementary file 1 [file cancers-13-00809-s001.pdf]

Supplementary Materials: The Florida Pancreas Collaborative Next-Generation Biobank: Infrastructure to Reduce Disparities and Improve Survival for a Diverse Cohort of Patients with Pancreatic Cancer

Jennifer B. Permuth, Kaleena B. Dezsi, Shraddha Vyas, Karla N. Ali, Toni L. Basinski, Ovie A. Utuama, Jason W. Denbo, Jason Klapman, Aamir Dam, Estrella Carballido, Dae Won Kim, Jose M. Pimiento, Benjamin D. Powers, Amy K. Otto, Jung W. Choi, Dung-Tsa Chen, Jamie K. Teer, Francisca Beato, Alina Ward, Elena M. Cortizas, Suzanne Y. Whisner, Iverson E. Williams, Andrea N. Riner, Kenneth Tardif, Vic Velanovich, Andreas Karachristos, Wade G. Douglas, Adrian Legaspi, Bassan J. Allan, Kenneth Meredith, Manual A. Molina-Vega, Philip Bao, Jamii St. Julien, Kevin L. Huguet, Lee Green, Folakemi T. Odedina, Nagi B. Kumar, Vani N. Simmons, Thomas J. George, Susan T. Vadaparampil, Pamela J. Hodul, J. Pablo Arnoletti, Ziad T. Awad, Debashish Bose, Kun Jiang, Barbara A. Centeno, Clement K. Gwede, Mokenge Malafa, Sarah M. Judge, Andrew R. Judge, Daniel Jeong, Mark Bloomston, Nipun B. Merchant, Jason B. Fleming, Jose G. Trevino and on behalf of the Florida Pancreas Collaborative

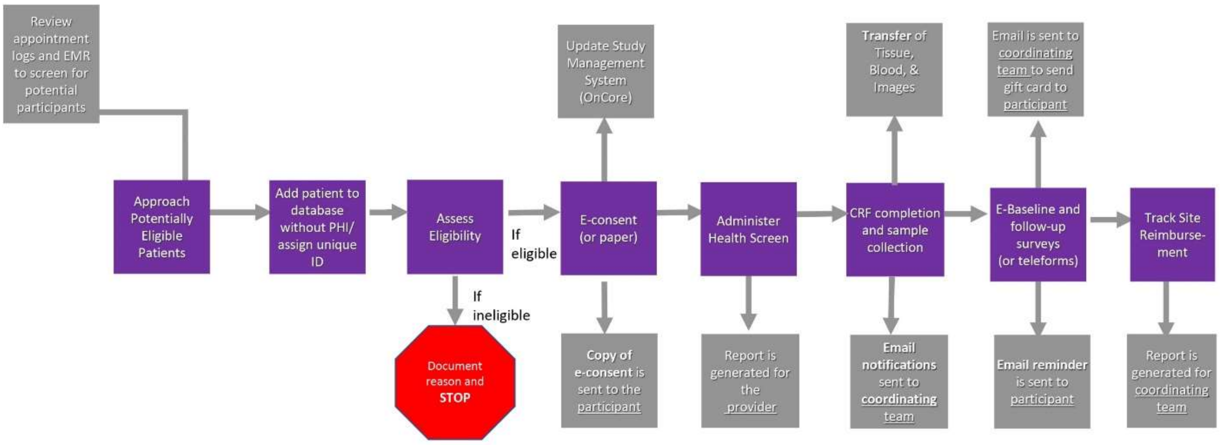

Figure S1. Workflow of Main Study-related Tasks Accomplished with the Assistance of our Online Data Management/Engagement Platform. Workflow includes screening patients to recruit, registering their demographic information and language preference (English or Spanish), automatic assignment of a unique identification number (ID) which is distinct from their medical record numbers and has a three-letter site prefix followed by four numbers (i.e. MCC-1234), recording approach outcomes, assessment of eligibility, obtaining informed consent electronically or documenting paper consent, verification of contact information for the participant and at least one individual who can be contacted if the participant cannot be reached, tracking of the collection, processing, transfer, and storage of biospecimens and images, sending automated email notifications related to study tasks, administration of questionnaires/surveys, and report generation. Abbreviations: EMR = electronic medical record; PHI=protected health information; E=electronic; CRF = case report form.

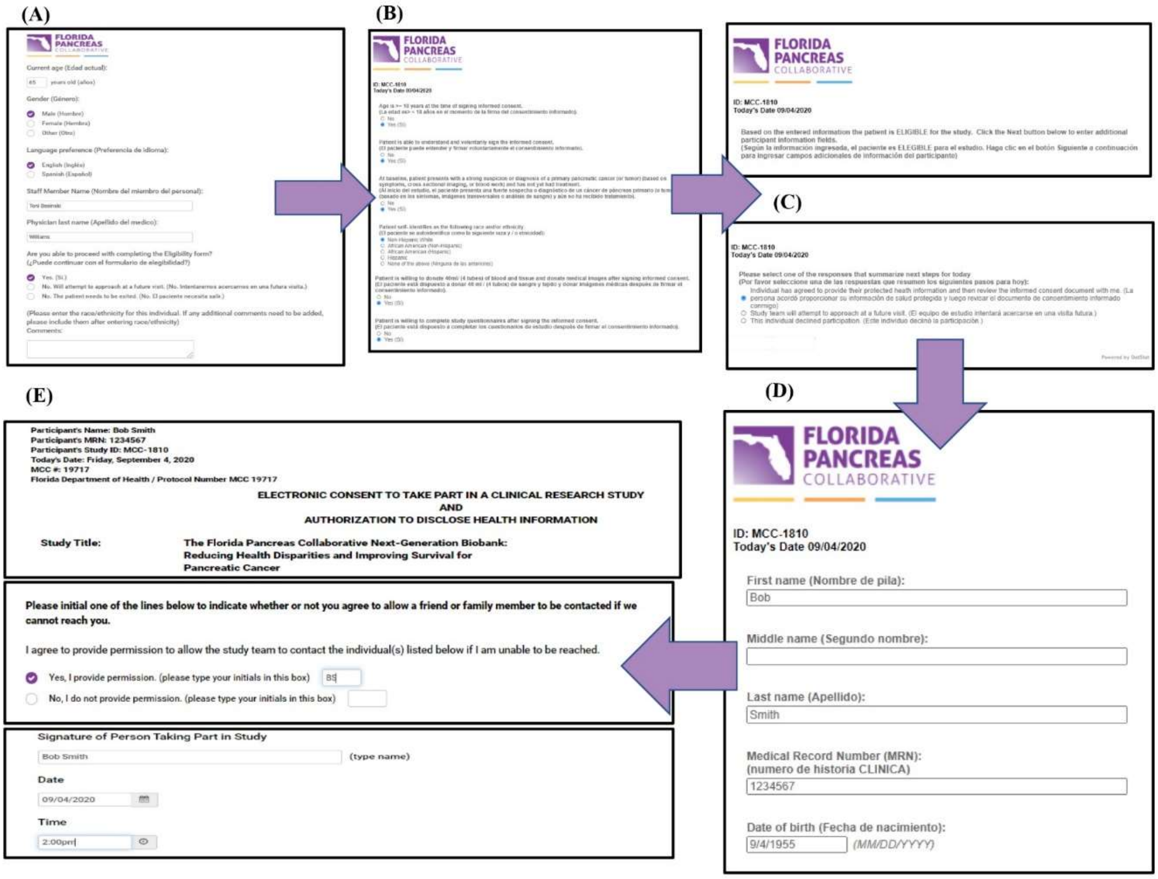

Figure S2. Workflow and Modules Represented in the Electronic-Consent (e-consent) Process. The E-Consent Process has an array of components that allow for more efficient recruitment. This process is deconstructed into multiple modules: (**A**) Add Participant Form, (**B**) Eligibility Form, (**C**) Approach Attempts, (**D**) Name and Contact Information Form, and (**E**) an informed consent form (ICF). Upon first screening a patient, the site coordinator completes the Add Participant form thereby collecting non-protected health information (PHI). Upon completion of that module, the coordinator is automatically directed to the Eligibility Form which then assesses the patient's eligibility through a series of questions. If eligible, the coordinator then chooses the next module depending on the outcome of the approach. If the patient agrees to consent, a Contact Information Module automatically populates and directly feeds all PHI into the E-Consent. Within the E-Consent, the patient electronically signs all portions of the document, including permission to contact a relative of their choosing if they cannot be reached during their study involvement. On the other hand, if the patient defers or declines, the coordinator is prompted to the Approach Attempts Module and Exit Module, respectively, where specific reasons for can be documented and pulled for future encounters.

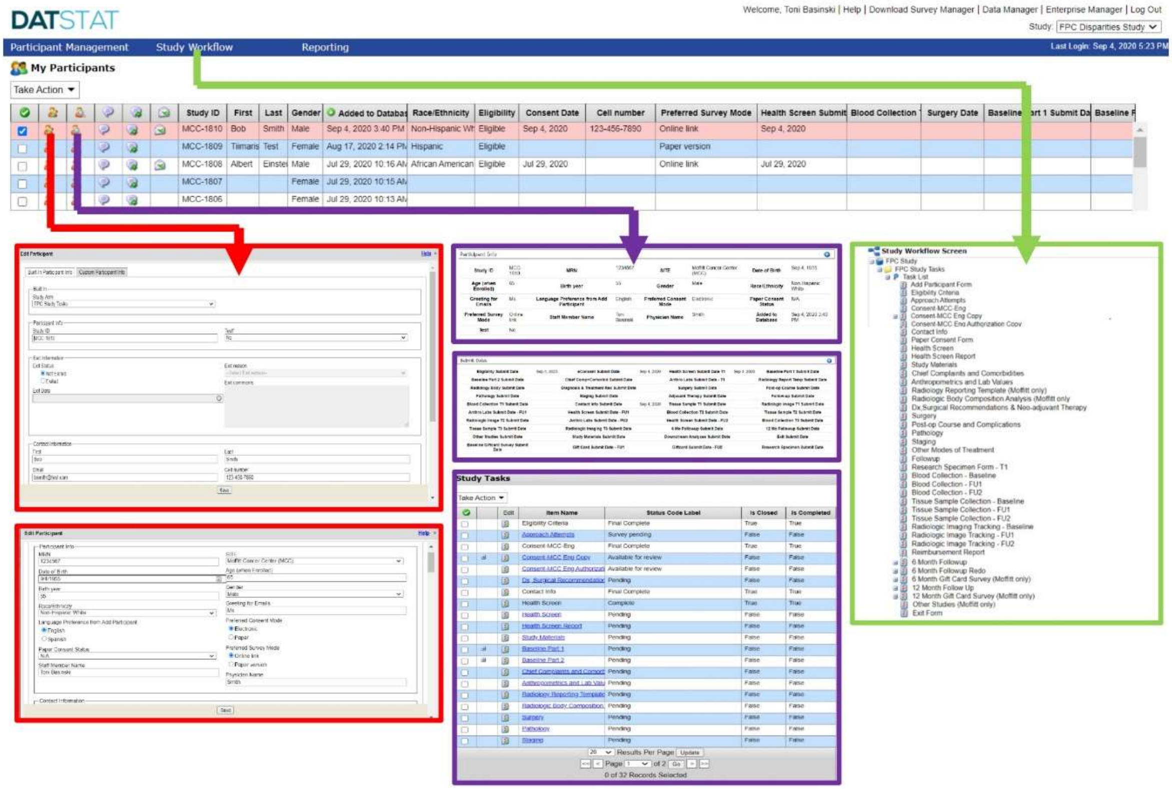

Figure S3. Examples of Data Management and Tracking Screens. The online data management platform includes customized views of all modules. Under the "Participant Management" tab, the study team can obtain a summary of all individuals that have been approached, consented, and/or exited along with key demographic fields in a convenient table format. This table format allows for a personalized snapshot of all participants dependent on what is being assessed by the coordinator. For instance, in this example the participants are arranged chronologically by consent dates, with the most recent consent appearing at the top. Each participant can then be seen individually in varying "profile" views. The first icon allows the coordinator quick access to edit all participant information including contact and study information (as seen in red). The second icon prompts for a unique profile view depicting all study information, form submit dates, as well as a table of Study Tasks (as seen in purple). The "Study Tasks" table allows the coordinator to quickly assess both module completion for data entry as well as biospecimen tracking at multiple timepoints. Finally, the coordinator can view all modules/case report forms in the "Study Workflow" view (as seen in green).

(A)

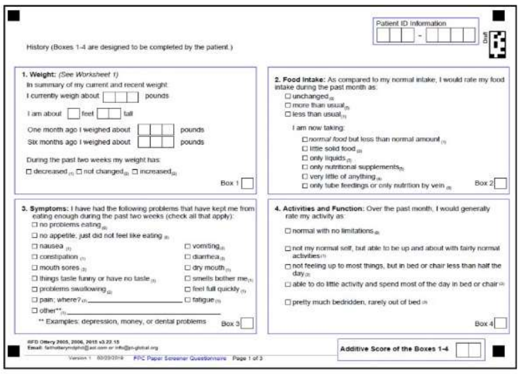

History (Boxes 1-4 are designed to be completed by the patient.)

Patient ID Information

Draft

1. Weight: *(See Worksheet 1)*
In summary of my current and recent weight:
I currently weigh about ☐☐☐ pounds
I am about ☐ feet ☐☐ tall
One month ago I weighed about ☐☐☐ pounds
Six months ago I weighed about ☐☐☐ pounds
During the past two weeks my weight has:
☐ decreased ☐ not changed ☐ increased
Box 1 ☐

2. Food Intake: As compared to my normal intake, I would rate my food intake during the past month as:
☐ unchanged
☐ more than usual
☐ less than usual
I am now taking:
☐ *normal food* but less than normal amount
☐ little solid food
☐ only liquids
☐ only nutritional supplements
☐ very little of anything
☐ only tube feedings or only nutrition by vein
Box 2 ☐

3. Symptoms: I have had the following problems that have kept me from eating enough during the past two weeks (check all that apply):
☐ no problems eating
☐ no appetite, just did not feel like eating
☐ nausea ☐ vomiting
☐ constipation ☐ diarrhea
☐ mouth sores ☐ dry mouth
☐ things taste funny or have no taste ☐ smells bother me
☐ problems swallowing ☐ feel full quickly
☐ pain; where? ☐ fatigue
☐ other**
** Examples: depression, money, or dental problems
Box 3 ☐

4. Activities and Function: Over the past month, I would generally rate my activity as:
☐ normal with no limitations
☐ not my normal self, but able to be up and about with fairly normal activities
☐ not feeling up to most things, but in bed or chair less than half the day
☐ able to do little activity and spend most of the day in bed or chair
☐ pretty much bedridden, rarely out of bed
Box 4 ☐

Additive Score of the Boxes 1-4 ☐☐

PFD Ottery 2005, 2006, 2015 v3.22.15
Email: [illegible] or info@pt-global.org
Version 1 02/23/2018 FPC Paper Screener Questionnaire Page 1 of 3

(B)

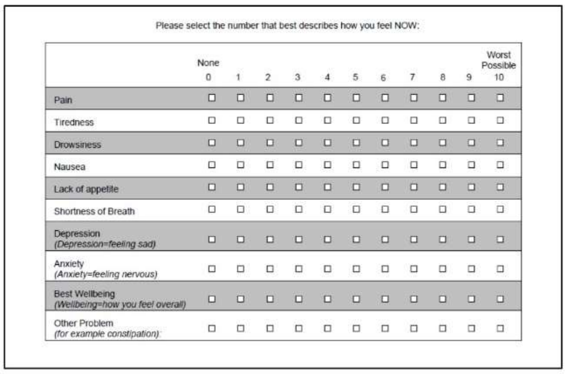

Please select the number that best describes how you feel NOW:

	None 0	1	2	3	4	5	6	7	8	9	Worst Possible 10
Pain	☐	☐	☐	☐	☐	☐	☐	☐	☐	☐	☐
Tiredness	☐	☐	☐	☐	☐	☐	☐	☐	☐	☐	☐
Drowsiness	☐	☐	☐	☐	☐	☐	☐	☐	☐	☐	☐
Nausea	☐	☐	☐	☐	☐	☐	☐	☐	☐	☐	☐
Lack of appetite	☐	☐	☐	☐	☐	☐	☐	☐	☐	☐	☐
Shortness of Breath	☐	☐	☐	☐	☐	☐	☐	☐	☐	☐	☐
Depression *(Depression=feeling sad)*	☐	☐	☐	☐	☐	☐	☐	☐	☐	☐	☐
Anxiety *(Anxiety=feeling nervous)*	☐	☐	☐	☐	☐	☐	☐	☐	☐	☐	☐
Best Wellbeing *(Wellbeing=how you feel overall)*	☐	☐	☐	☐	☐	☐	☐	☐	☐	☐	☐
Other Problem *(for example constipation):*	☐	☐	☐	☐	☐	☐	☐	☐	☐	☐	☐

(C)

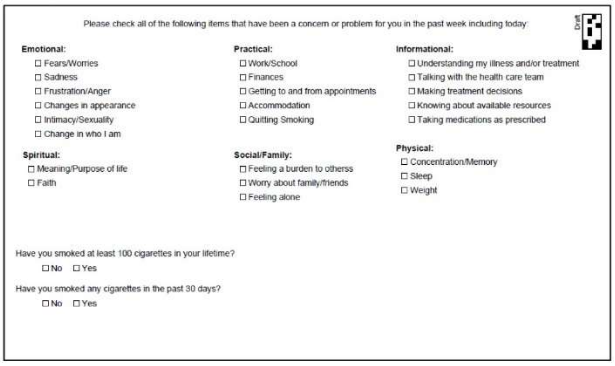

Please check all of the following items that have been a concern or problem for you in the past week including today:

Draft

Emotional:
☐ Fears/Worries
☐ Sadness
☐ Frustration/Anger
☐ Changes in appearance
☐ Intimacy/Sexuality
☐ Change in who I am

Practical:
☐ Work/School
☐ Finances
☐ Getting to and from appointments
☐ Accommodation
☐ Quitting Smoking

Informational:
☐ Understanding my illness and/or treatment
☐ Talking with the health care team
☐ Making treatment decisions
☐ Knowing about available resources
☐ Taking medications as prescribed

Spiritual:
☐ Meaning/Purpose of life
☐ Faith

Social/Family:
☐ Feeling a burden to otherss
☐ Worry about family/friends
☐ Feeling alone

Physical:
☐ Concentration/Memory
☐ Sleep
☐ Weight

Have you smoked at least 100 cigarettes in your lifetime?
☐ No ☐ Yes

Have you smoked any cigarettes in the past 30 days?
☐ No ☐ Yes

Figure S4. Florida Pancreas Collaborative Health Screen Instrument. This instrument comprises (**A**)the abridged version of the Patient-Generated Subjective Global Assessment (aPG-SGA), (**B**) a revised version of the Edmonton Symptom Assessment System (ESAS-r), and (C)the Canadian Problem Checklist.

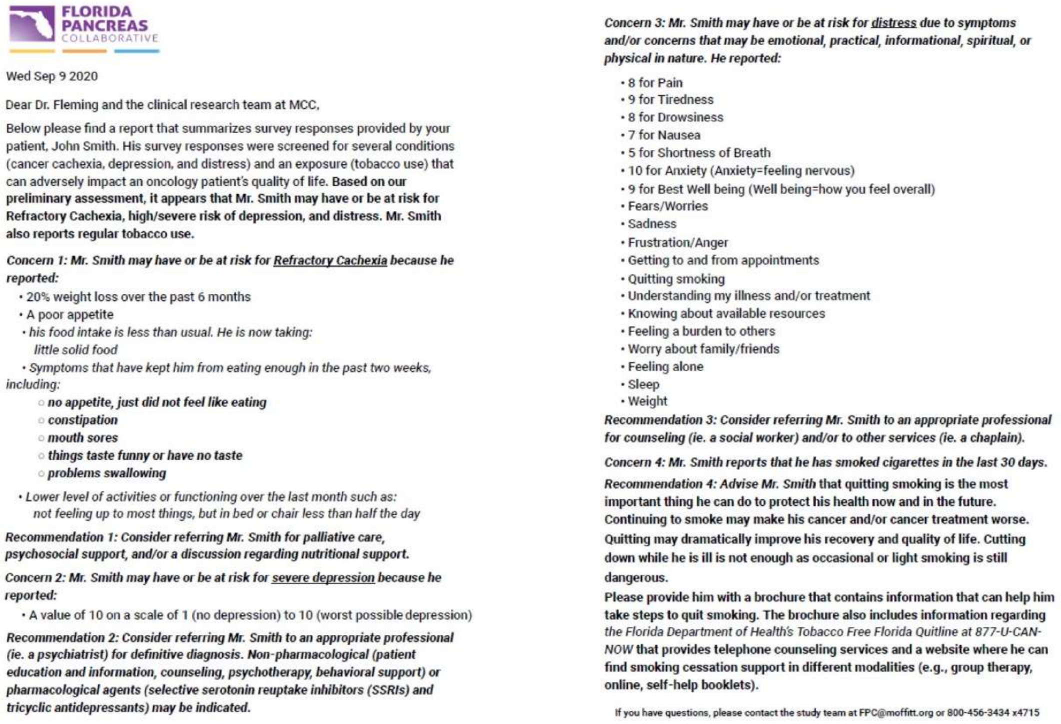

FLORIDA PANCREAS COLLABORATIVE

Wed Sep 9 2020

Dear Dr. Fleming and the clinical research team at MCC,

Below please find a report that summarizes survey responses provided by your patient, John Smith. His survey responses were screened for several conditions (cancer cachexia, depression, and distress) and an exposure (tobacco use) that can adversely impact an oncology patient's quality of life. **Based on our preliminary assessment, it appears that Mr. Smith may have or be at risk for Refractory Cachexia, high/severe risk of depression, and distress. Mr. Smith also reports regular tobacco use.**

Concern 1: Mr. Smith may have or be at risk for <u>Refractory Cachexia</u> because he reported:

- 20% weight loss over the past 6 months
- A poor appetite
- *his food intake is less than usual. He is now taking:*
  little solid food
- *Symptoms that have kept him from eating enough in the past two weeks, including:*
  - ***no appetite, just did not feel like eating***
  - ***constipation***
  - ***mouth sores***
  - ***things taste funny or have no taste***
  - ***problems swallowing***
- *Lower level of activities or functioning over the last month such as:*
  not feeling up to most things, but in bed or chair less than half the day

Recommendation 1: Consider referring Mr. Smith for palliative care, psychosocial support, and/or a discussion regarding nutritional support.

Concern 2: Mr. Smith may have or be at risk for <u>severe depression</u> because he reported:

- A value of 10 on a scale of 1 (no depression) to 10 (worst possible depression)

Recommendation 2: Consider referring Mr. Smith to an appropriate professional (ie. a psychiatrist) for definitive diagnosis. Non-pharmacological (patient education and information, counseling, psychotherapy, behavioral support) or pharmacological agents (selective serotonin reuptake inhibitors (SSRIs) and tricyclic antidepressants) may be indicated.

Concern 3: Mr. Smith may have or be at risk for <u>distress</u> due to symptoms and/or concerns that may be emotional, practical, informational, spiritual, or physical in nature. He reported:

- 8 for Pain
- 9 for Tiredness
- 8 for Drowsiness
- 7 for Nausea
- 5 for Shortness of Breath
- 10 for Anxiety (Anxiety=feeling nervous)
- 9 for Best Well being (Well being=how you feel overall)
- Fears/Worries
- Sadness
- Frustration/Anger
- Getting to and from appointments
- Quitting smoking
- Understanding my illness and/or treatment
- Knowing about available resources
- Feeling a burden to others
- Worry about family/friends
- Feeling alone
- Sleep
- Weight

Recommendation 3: Consider referring Mr. Smith to an appropriate professional for counseling (ie. a social worker) and/or to other services (ie. a chaplain).

Concern 4: Mr. Smith reports that he has smoked cigarettes in the last 30 days.

Recommendation 4: Advise Mr. Smith **that quitting smoking is the most important thing he can do to protect his health now and in the future. Continuing to smoke may make his cancer and/or cancer treatment worse. Quitting may dramatically improve his recovery and quality of life. Cutting down while he is ill is not enough as occasional or light smoking is still dangerous.**

Please provide him with a brochure that contains information that can help him take steps to quit smoking. The brochure also includes information regarding *the Florida Department of Health's Tobacco Free Florida Quitline at 877-U-CAN-NOW* **that provides telephone counseling services and a website where he can find smoking cessation support in different modalities (e.g., group therapy, online, self-help booklets).**

If you have questions, please contact the study team at FPC@moffitt.org or 800-456-3434 x4715

Figure 5. Example of a Health Screen Report Obtained at Baseline.

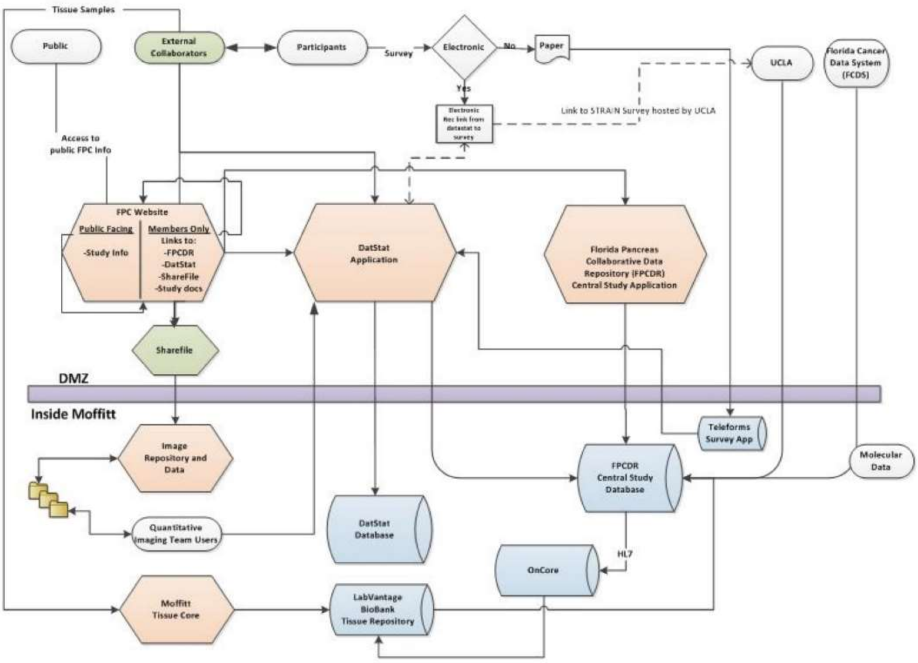

Figure S6. Florida Pancreas Collaborative Data Repository (FPCDR) Infrastructure..

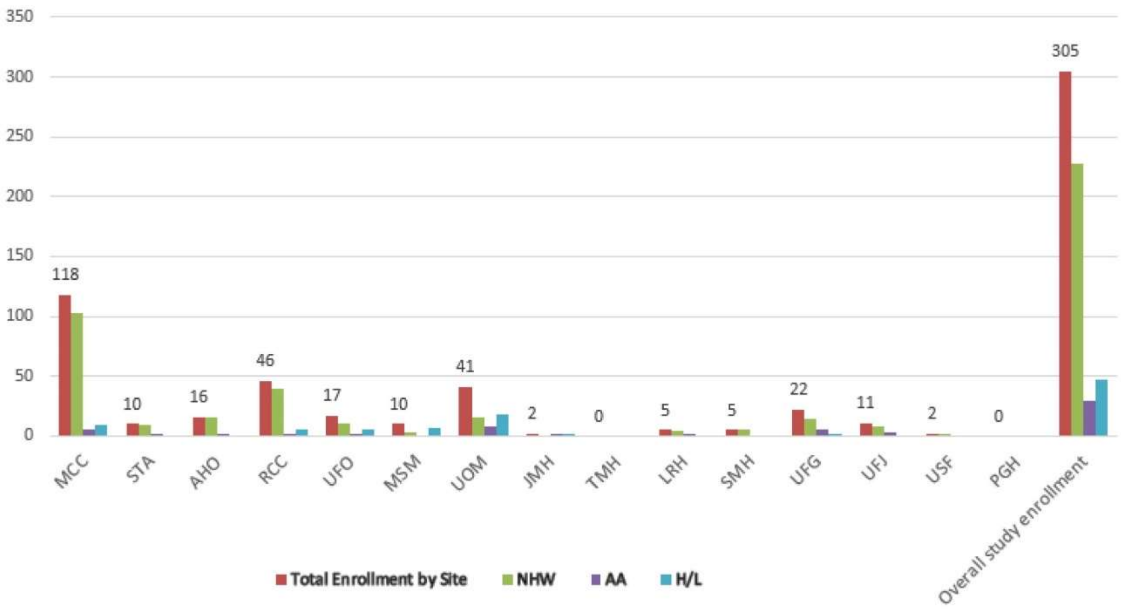

Figure S7. Total number of participants enrolled in the Florida Pancreas Collaborative, by study site and race/ethnicity. Note: These numbers reflect enrollment through August 31, 2020.

Table S1. Sensitivity analysis comparing demographic and clinical characteristics by follow-up status.

		Exited		
**Variable**	**All participants (*n* = 305)**	**No (*n* = 258)**	**Yes (*n* = 47)**	***P*-Value**
Age (years), mean (+/− SD)	68 (10.6)	68 (10.6)	70 (10.5)	0.3190

Gender, n (%)				
Female	161 (52.8)	136 (52.7)	25 (53.2)	0.9518
Male	144 (47.2)	122 (47.3)	22 (46.8)	
Race/Ethnicity†, n (%)				
African American (AA)	30 (9.8)	27 (10.5)	3 (6.4)	0.4741
Hispanic/Latinx (H/L)	47 (15.4)	42 (16.3)	5 (10.6)	
Non-Hispanic White (NHW)	228 (74.8)	189 (73.3)	39 (83.0)	
Education Level†, n (%)				
High school or GED	46 (31.1)	39 (31.7)	7 (28.0)	0.9047
College	65 (43.9)	54 (43.9)	11 (44.0)	
Post graduate	37 (25.0)	30 (24.4)	7 (28.0)	
Data not yet availableɸ	157	135	22	
Income Level†, n (%)				
Below $40k	38 (26.0)	28 (23.1)	10 (40.0)	0.1957
$40k-100k	42 (28.8)	37 (30.6)	5 (20.0)	
100k and above	34 (23.3)	27 (22.3)	7 (28.0)	
Information not provided by Participant	32 (21.9)	29 (24.0)	3 (12.0)	
Data not yet availableɸ	159	137	22	
Health Insurance†, n (%)				
Insured	143 (97.9)	120 (99.2)	23 (92.0)	0.0760
Uninsured	3 (2.1)	1 (0.8)	2 (8.0)	
Data not yet availableɸ	159	137	22	
Marital Status†, n (%)				
Not married	38 (26.0)	30 (24.8)	8 (32.0)	0.0858
Married	107 (73.2)	91 (75.2)	16 (64.0)	
Information not provided by Participant	1 (0.8)	0 (0.0)	1 (4.0)	
Data not yet availableɸ	159	137	22	
Family History of Pancreatic Cancer†, n (%)				
No	87 (67.4)	75 (69.4)	12 (57.1)	0.0779
Yes	16 (12.4)	15 (13.9)	1 (4.8)	
Participant does not know	26 (20.2)	18 (16.7)	8 (38.1)	
Data not yet availableɸ	176	150	26	
Distress‡, n (%)				
No	36 (12.4)	35 (14.3)	1 (2.2)	0.025
Yes	255 (87.6)	210 (85.7)	45 (97.8)	
Data not yet availableɸ	14	13	1	
Depression‡, n (%)				
No	189 (64.9)	157 (64.1)	32 (69.6)	0.5707
Mild depression	43 (14.8)	39 (15.9)	4 (8.7)	
Moderate depression	40 (13.8)	34 (13.9)	6 (13.0)	
Severe depression	19 (6.5)	15 (6.1)	4 (8.7)	
Data not yet availableɸ	14	13	1	

Smoking status†‡, n (%)				
No	129 (44.3)	109 (44.3)	20 (44.4)	0.4935
Former smoker	127 (43.6)	105 (42.7)	22 (48.9)	
Current smoker	35 (12.1)	32 (13.0)	3 (6.7)	
Data not yet availableϕ	14	12	2	
Marijuana status†, n (%)				
No	97 (71.9)	76 (68.5)	21 (87.5)	0.2575
Former user	25 (18.5)	23 (20.7)	2 (8.3)	
Current user	13 (9.6)	12 (10.8)	1 (4.2)	
Data not yet availableϕ	170	147	23	
Abdominal Pain¶, n (%)				
No	78 (38.8)	72 (41.4)	6 (22.2)	0.1512
Yes	100 (49.8)	83 (47.7)	17 (63.0)	
Information unavailble in EMR	23 (11.4)	19 (10.9)	4 (14.8)	
Data not yet availableϕ	104	84	20	
Fatigue¶, n (%)				
No	148 (52.5)	125 (52.3)	23 (53.5)	0.1754
Yes	98 (34.7)	80 (33.5)	18 (41.9)	
Information unavailble in EMR	36 (12.8)	34 (14.2)	2 (4.6)	
Data not yet availableϕ	23	19	4	
GI Bleeding¶, n (%)				
No	217 (77.0)	181 (75.7)	36 (83.7)	0.0269
Yes	7 (2.5)	4 (1.7)	3 (7.0)	
Information unavailble in EMR	58 (20.5)	54 (22.6)	4 (9.3)	
Data not yet available	23	19	4	
Jaundice¶, n (%)				
No	178 (62.9)	152 (63.3)	26 (60.5)	0.1893
Yes	66 (23.3)	52 (21.7)	14 (32.5)	
Information unavailble in EMR	39 (13.8)	36 (15.0)	3 (7.0)	
Data not yet availableϕ	22	18	4	
Weight Loss More Than 5%¶, n (%)				
No	133 (47.4)	115 (48.3)	18 (41.9)	0.7094
Yes	115 (40.9)	95 (39.9)	20 (46.5)	
Information unavailble in EMR	33 (11.7)	28 (11.8)	5 (11.6)	
Data not yet availableϕ	24	20	4	
Charlsons Comorbidity Index, n (%)				
0	164 (57.7)	142 (58.9)	22 (51.2)	0.1975
<=2	101 (35.6)	81 (33.6)	20 (46.5)	
>=3	19 (6.7)	18 (7.5)	1 (2.3)	
Data not yet availableϕ	21	17	4	

Personal History of Diabetes†¶, n (%)				
No	195 (68.4)	167 (69.0)	28 (65.1)	0.6129
Yes	90 (31.6)	75 (31.0)	15 (34.9)	
Data not yet availableɸ	20	16	4	
Personal History of Pancreatitis†¶, n (%)				
No	180 (79.6)	158 (81.0)	22 (71.0)	0.1964
Yes	46 (20.4)	37 (19.0)	9 (29.0)	
Data not yet availableɸ	79	63	16	
Cachexia‡¶, n (%)				
refractory cachexia	10 (3.8)	5 (2.3)	5 (11.6)	0.0039
cachexia	76 (29.1)	59 (27.0)	17 (39.5)	
pre-cachexia	26 (10.0)	25 (11.5)	1 (2.4)	
non cachectic	149 (57.1)	129 (59.2)	20 (46.5)	
missing	44	40	4	
Body Mass Index (kg/m2)¶ n, mean (SD)	281, 27 (5.5)	235, 27 (5.5)	46, 26 (5.5)	0.1791
Waist Circumference,¶ n, mean (SD)	231, 40 (12.7)	191, 40 (12.7)	40, 40 (12.7)	0.9476
Histology¶, n (%)				
Pancreatic Ductal Adenocarcinoma (PDAC)	183 (61.4)	154 (61.1)	29 (63.0)	0.0064
Pancreatic Neuroendocrine Tumor (PNET)	35 (11.7)	33 (13.1)	2 (4.4)	
Intraductal Papillary Mucinous Neoplasm (IPMN)	35 (11.7)	33 (13.1)	2 (4.4)	
Mucinous Cystic Neoplasm (MCN)	6 (2.0)	6 (2.4)	0 (0.0)	
Other§	39 (13.1)	26 (10.3)	13 (28.2)	
Data not yet availableɸ	7	6	1	
Surgical Resection Attempted¶, n (%)				
No	146 (47.9)	116 (45.0)	30 (63.8)	
Yes	159 (52.1)	142 (55.0)	17 (36.2)	
Location of Tumor¶, n (%)				
Body	16 (14.0)	16 (14.0)	0 (0.0)	0.5527
Diffuse	17 (14.9)	15 (13.2)	2 (20.0)	
Head	65 (57.0)	60 (52.6)	5 (50.0)	
Tail	13 (11.4)	12 (10.5)	1 (10.0)	
Other	13 (11.4)	11 (9.7)	2 (20.0)	
Data not yet availableɸ	181	144	37	
Stage¶, n (%)				

Stage 0	22 (14.1)	20 (14.8)	2 (13.3)	<.0001
Stage I/II	87 (55.8)	85 (63.0)	2 (13.3)	
Stage III/IV	41 (26.3)	30 (22.2)	11 (73.3)	
Data not yet availableϕ	155	123	32	
Grade Exocrine Pancreatic Tumor¶¥, n (%)				
Well differentiated	7 (8.0)	7 (8.7)	0 (0.0)	0.0804
Moderately differentiated	29 (32.9)	29 (36.3)	0 (0.0)	
Poorly differentiated	21 (23.9)	18 (22.5)	3 (37.5)	
Grade undetermined	31 (35.2)	26 (32.5)	5 (62.5)	
Data not yet availableϕ	136	113	23	
Grade IPMN¶, n (%)				
Low grade	12 (34.3)	12 (36.4)	0 (0.0)	0.6975
Borderline	2 (5.7)	2 (6.1)	0 (0.0)	
Carcinoma-in-situ	5 (14.3)	5 (15.1)	0 (0.0)	
Invasive carcinoma	1 (2.9)	1 (3.0)	0 (0.0)	
Unknown	15 (42.9)	13 (39.4)	2 (100.0)	
Positive Lymph Nodes¶, n (%)				
No	110 (94.8)	100 (95.2)	10 (90.9)	0.4576
Yes	6 (5.2)	5 (4.8)	1 (9.1)	
Data not yet availableϕ	189	153	36	

†: Data available from partially-or fully-completed baseline questionnaires at time of analysis. ϕ: Data not yet available. Additional data will be included in future analyses when entered into the DatStat system. ‡: Data available from the health screen questionnaire at time of analysis. ¶: Data available from case report forms (CRFs) at time of analysis. §: The 'Other' category includes benign and malignant tumors of pancreatic, liver and bile duct, renal, adrenal gland, lymph node, and unclassified origin. ¥: The grade for exocrine tumor has been restricted to PDAC, IPMN, and MCN (n = 224).
